# Investigation of anti-nociceptive, anti-inflammatory potential and ADMET studies of pure compounds isolated from *Isodon rugosus* Wall. ex Benth

**DOI:** 10.3389/fphar.2024.1328128

**Published:** 2024-02-13

**Authors:** Osama M. Alshehri, Anwar Zeb, Syed Muhammad Mukarram Shah, Mater H. Mahnashi, Saeed Ahmed Asiri, Omaish Alqahtani, Abdul Sadiq, Muhammad Ibrar, Saleh Alshamrani, Muhammad Saeed Jan

**Affiliations:** ^1^ Department of Clinical Laboratory Sciences, College of Applied Medical Sciences, Najran University, Najran, Saudi Arabia; ^2^ Department of Pharmacy, University of Swabi, Swabi, Pakistan; ^3^ Department of pharmaceutical chemistry, College of pharmacy, Najran University, Najran, Saudi Arabia; ^4^ Department of Clinical Laboratory Sciences, Faculty of Applied Medical Sciences, Najran University, Najran, Saudi Arabia; ^5^ Department of Pharmacognosy, College of Pharmacy, Najran University, Najran, Saudi Arabia; ^6^ Department of Pharmacy, Univeristy of Malakand, Chakdara, Pakistan; ^7^ Department of Pharmacy, Bacha Khan University, Charsadda, Pakistan

**Keywords:** *Isodon rugosus*, antinociception, anti-inflammatory, opioid receptors, adrenergic receptors

## Abstract

The strong ethnopharmacological utilization of *Isodon rugosus* Wall. Ex. Benth is evident in the treatment of several types of pain and inflammation, including toothache, earache, abdominal pain, gastric pain, and generalized body pain and inflammation. Based on this background, the antinociceptive effects of the crude extract, various fractions, and essential oil have been reported previously. In this research work, we isolate and characterize pure bioactive compounds from *I. rugosus* and evaluate possible mechanisms using various *in vivo* and *in vitro* models. The pure compounds were analyzed for analgesic and anti-inflammatory activities through various assays. The column chromatography of the chloroform fraction of *I. rugosus* led to the identification of two pure compounds, i.e., 1 and 2. Compound 1 demonstrated notable inhibition (62% writhing inhibition, 72.77% COX-2 inhibition, and 76.97% 5-LOX inhibition) and anti-inflammatory potential (>50% paw edema inhibition at various intervals). The possible mechanism involved in antinociception was considered primarily, a concept that has already been elucidated through the application of naloxone (an antagonist of opioid receptors). The involvement of adrenergic receptors was investigated using a hot plate model (an adrenergic receptor antagonist). The strong ethnomedicinal analgesic background of *I. rugosus*, supported by previous reports and current observations, leads to the conclusion that *I. rugosus* is a potential source of antinociceptive and anti-inflammatory bioactive compounds. It may be concluded from the results that the isolated analgesic compounds of *I. rugosus* may be a possible alternative remedy for pain and inflammation management with admirable efficacy and safety profiles.

## Graphical Abstract

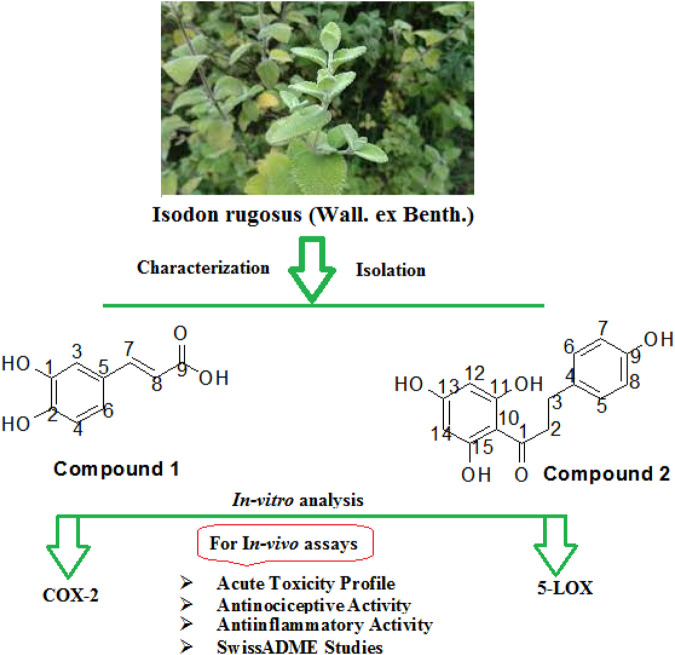

## 1 Introduction

Nowadays, natural products play a very significant role in managing various types of diseases. Researchers should take advantage of utilizing the different natural resources for the benefit of mankind (Shah et al., 2012). Different drugs have been discovered by researchers for their benefits in routine life (Patwardhan et al., 2004). In this modern era, we are facing many emerging diseases that lead to an increasing human mortality rate day by day (Shah et al., 2014). To overcome this bitter fact and minimize different threats to human health, scientists have to work to find a better solution (Sadiq et al., 2018). In this regard, researchers have been focused on the development of new medicinal compounds to treat different health-related problems (Zahoor et al., 2018). Shockingly, only two percent of the 300,000 plant species have undergone scientific investigation (Awai et al., 2001). Recently, a large number of bioactive compounds with medicinal significance have been isolated from medicinal plants using a range of scientific techniques aligned with their ethno-medicinal applications (Zafar et al., 2019). Natural drugs obtained from medicinal plants have great importance in solving health issues as they have fewer side effects and economic benefits (Jan et al., 2020). Many diseases affect human life to a drastic level, such as myocardial infarction, angina, trauma, and surgery that accompanies pain (Stanik-Hutt et al., 2001; Khan et al., 2022). A literature survey revealed that different methods have been employed for the management of pain and inflammation for centuries. In ancient times, people believed that pain was a divine punishment (Rye et al., 2000). Cupping therapy, acupuncture, or massage therapy was applied to relieve the pain (Munir et al., 2020). For the management of pain and inflammation, various NSAIDs are available, but they may cause various side effects, such as bleeding, ulcers, difficulty in urinating, and seizures (Alam et al., 2020; Javed et al., 2021). In this regard, there is a need for herb-derived compounds that have minimum side effects and excellent efficacy and economic status (Houghton, 1995; Abbas et al., 2022). The formation of pain and inflammatory mediators starts with lipid peroxidation, wherein arachidonic acid is formed; subsequently, the conversion of arachidonic acid into prostaglandins and leukotrienes occurs through different pathways with the help of COX and LOX. Similarly, the inhibition of COX and LOX inhibits the formation of prostaglandins and leukotrienes, thereby relieving and preventing pain and inflammation (Martel-Pelletier et al., 2003; Ahsan et al., 2023).

*Isodon rugosus* is a prominent member of the family Labiatae. Its bark is used ethnomedicinally in relieving general body pain and treating dysentery (Mahnashi et al., 2021). The dried leaves are put in the mouth to cure toothache (Akhtar et al., 2013), and 1‒2 drops of the fresh leaf extract are used for earache. It has additionally been reported to relieve gastric and abdominal discomfort (Ahmad et al., 2014).

Ethnomedicinally, it is also used in the management of conditions such as hypertension, pyrexia, and rheumatoid arthritis, as an antiemetic and antispasmodic, and for its antimicrobial and antifungal activities (Khan and Khatoon, 2007; Adnan et al., 2012; Janbaz et al., 2014; Shuaib et al., 2014). Considering the ethnomedicinal applications of *I. rugosus*, the present research work is carried out to determine the analgesic and anti-inflammatory effects of the isolated compounds from *I. rugosus* through both *in vivo* and *in vitro* investigations, along with elucidating the underlying mechanisms.

## 2 Materials and methods

### 2.1 Plant collection and extraction

The fresh aerial components from the plant were collected in July from northern areas of KPK, Pakistan, and were identified by the plant taxonomist Dr. Ali Hazrat, at the University of Malakand (KPK), Pakistan (voucher number 1016AZ). The plant was washed, shade-dried, and ground into powder. The plant powder, weighing 8 kg, was macerated in methanol (80%) for three weeks. Filtration was done using a filter paper, and a rotary evaporator was used for the evaporation of the solvent and collection of the extract under reduced pressure at 40°C (Ahmad et al., 2015; Kamal et al., 2015). A brownish-green semi-solid methanolic extract (600 g) was obtained.

### 2.2 Fractionation and isolation of compounds

Fractionation was performed by adding 500 ml of distilled water to the crude methanolic extract in a separating funnel, followed by the addition of chloroform (500 ml). It was shaken at different intervals and allowed to stand to obtain two distinct layers. The same process was repeated three times for the separation of chloroform. The collected chloroform was concentrated using a rotary evaporator at reduced pressure. A semi-solid form of the chloroform extract, weighing 44 g, was obtained. Fractionation was done based on increasing polarity. As per our earlier work, we subjected the chloroform fraction to column-based chromatography for the separation of phytochemicals. We investigated the elution of compounds from the selected fraction on pre-coated silica gel TLC plates via the rising polarity elusion approaches. We started the chromatographic method with simply pure n-hexane initially and then slowly increased the polarity via the addition of 2% ethyl acetate each time. After each addition of ethyl acetate, TLC was visualized, and the polarity was adjusted correspondingly. Then, depending on the amount of fractions, we took a large column packed with flash silica slurry with a suitable fraction. Elution started with that of the non-polar n-hexane and gradually rose in polarity with ethyl acetate. Partially pure fractions, approximately up to 80%, were produced. Then, this was again loaded to pencil columns. The columns were once again eluted with n-hexane and chloroform solvent solutions for targeted component purification. The phytochemicals (1 and 2) were extracted from the chloroform bioactive fraction. Compounds 1 and 2 were purified, yielding 251 mg and 277 mg, respectively (Ayaz et al., 2014a; Ayaz et al., 2014b).

### 2.3 Characterization of isolated compounds

Various spectroscopic techniques, such as ^1^H NMR, ^13^C NMR, and MS, were employed for the characterization and identification of the pure isolated compounds.

### 2.4 Lipoxygenase inhibitory activity

The test samples of *I. rugosus* were evaluated for lipoxygenase inhibitory activity using the following procedure (Ulusu et al., 2002). The enzyme solution with a concentration of 10,000 U/ml was prepared. The test samples were prepared with different concentrations, i.e., 1000, 500, 250, 125, and 62.5 μg/ml. The positive control used was zileuton. A solution (80 mM) of linoleic acid (substrate) was made. Likewise, a 50 mM phosphate buffer solution with a pH of 6.3 was prepared. The substrate, enzyme, and buffer were combined in equal proportions, each in a 2 ml volume. An UV-visible spectrophotometer was used to determine the percent enzyme inhibition at 234 nm. The inhibition percentage was determined by comparing the absorbance of the test samples with that of the positive control.

### 2.5 Cyclooxygenase-2 inhibitory activity

The COX-2 assay was performed to investigate the anti-nociceptive activity using the previously reported procedure (Burnett et al., 2007; Javed et al., 2022). An enzyme solution with a concentration of 300 U/ml was prepared and activated using ice for 5 min with a 50 µL cofactor solution containing glutathione (0.9 mM), TMPD (*N*,*N*,*N*,*N*_tetramethyl-p-phenylenediaminedihydrochloride) (0.24 mM), and hematin (1 mM) in Tris buffer (0.1 M) with pH 8. The enzyme solution (60 µL) and test samples (20 µL) with various concentrations were incubated at room temperature for 5 minutes. Celecoxib was used as a positive control in the current assay. The absorbance of the samples was measured at 570 nm after the samples had been incubated for 5 min. Arachidonic acid (30 mM, 20 µL) was added to initiate the reaction. The percent inhibition was calculated from the absorbance value per unit time.

### 2.6 Experimental animals and acute toxicity tests

An anti-nociceptive assessment was performed on Swiss albino mice of both sexes, sourced from the National Institute of Health, Islamabad, Pakistan. All animal models were employed under the authorization of the Ethical Committee of the Department of Pharmacy, University of Swabi, Pakistan, with the ethical approval number UOS-05/522 in accordance with the 2008 Animals Bye-Laws (Scientific Procedures, Issue-I). Mice of either sex were divided into different groups, with five mice in each group. The test samples were carefully administered orally, with doses of 250, 500, 1000, and 2000 mg/kg. Following the administration of doses, the mice were monitored for the subsequent 72 h to detect any allergic reaction or unusual behavior (Council, 1996; Hosseinzadeh et al., 2000).

### 2.7 Analgesic activity

#### 2.7.1 Acetic acid-induced writhing test

*I. rugosus* was evaluated for its analgesic potential through an acetic acid-induced writhing test. Oral administration of the test samples was carried out using specified doses. After 30 min of interval, 10 ml/kg of acetic acid (0.6%) was injected into the mice. Group I served as the control and received 0.5% Tween-80 (3 ml/kg), group II received diclofenac sodium (10 mg/kg), and groups III and IV received test compounds. The count of writhes was recorded between 5 and 30 min, following the *i/p* injection of acetic acid (Franzotti et al., 2000).

#### 2.7.2 Formalin test

The analgesic activity of *I. rugosus* was assessed through a formalin-induced licking test. The test samples were administered orally at specified doses. All the animals received formalin subcutaneously into the plantar surface of the paw at a dose of 2.5% (20 µL) after 30 min. Group I (the negative control) received 0.5% Tween-80 (3 ml/kg), and group II was administered morphine (5 mg/kg), while the other groups (III and IV) were injected with test compounds. The total time spent licking and biting the injected paw was recorded for up to 30 min (Sulaiman et al., 2008).

#### 2.7.3 Hot plate test

A hot plate was used for the evaluation of the analgesic activity of *I. rugosus* by following the reported procedure (Muhammad et al., 2012). The analgesic effects of the isolated compounds were measured using the hot plate method at a monitored temperature of 55 ± 0.2°C. The Swiss albino mice were positioned on the heated surface of the hot plate, and the period from placement to the onset of licking their hind paws or starting jump movements was noted as the response latency (thermal reactions). The test compounds were injected intraperitoneally. Morphine was given at a dosage of 5 mg/kg for 30 min before starting the test. The animal models were checked for thermal reactions at various time intervals after sample administration.

#### 2.7.4 Opioid receptor involvement

The involvement of opioid receptors was verified by the pretreatment of test models with naloxone (5 mg/kg), administered 15 min before morphine and test compounds using the hot plate and the formalin test.

#### 2.7.5 Involvement of adrenergic receptors

To evaluate the adrenergic receptor involvement in the nociception mechanism (Naidu et al., 2003; Alshehri et al., 2022), yohimbine (*α*_2_ adrenergic antagonist) was administered to mice before the administration of clonidine (*α*_2_ agonist). The outcomes of the positive control and tested compounds were compared in animals pretreated with yohimbine. To confirm the adrenergic receptor involvement in the affected groups of animals, yohimbine (1 mg/kg) was administered as a form of pre-treatment through intravenous injection 15 min prior to the introduction of the tested compounds. Additionally, clonidine at a dose of 3 μg/kg was intravenously administered. The animals were then subjected to the hot plate method, as reported before.

### 2.8 *In vivo* anti-inflammatory activity

#### 2.8.1 Carrageenan-induced inflammation

The carrageenan-induced assay was utilized to evaluate the potential anti-inflammatory effects (Winter et al., 1963). The animals under study were divided into different groups. Prior to the experiment, the animals had only access to water. Diclofenac sodium at a dose of 50 mg/kg was used as the standard, while normal saline served as the negative control group. Additionally, the isolated compounds being tested were given 30 min before injecting 0.05 ml of 1% carrageenan into the sub-plantar region of the paw. To measure the volume changes in the paw, a digital plethysmometer was employed. Readings were taken before and after the carrageenan injection at hourly intervals over a total period of 5 h.

### 2.9 Pharmacokinetic studies

The concept of drug-like chemical space plays a key role in the development and selection of potential drug candidates. It refers to compounds possessing pharmacokinetic potentials suitable for successfully navigating human Phase I clinical trials (Bade et al., 2010). The physicochemical properties, water solubility, lipophilicity, pharmacokinetic profile, and medicinal chemistry of the compounds were predicted using the SwissADME database (Daina et al., 2017; Ahmad et al., 2021a). The database contained 2D structures that enabled the use of a string-based search. The simplified molecular-input line-entry system (SMILES) was used to represent the structures of various ligands.

### 2.10 Statistical data analysis

The test results are tabulated as the mean ± S.E.M. Significance in the percentage inhibition of different test samples was assessed through one-way ANOVA, followed by Bonferroni’s post-test analysis using GraphPad Prism software, where values of *p* < 0.05 were deemed significant. The IC_50_ values were calculated using the SPSS program.

## 3 Results

### 3.1 Isolated compounds and structural elucidation

**FIGURE 1:**
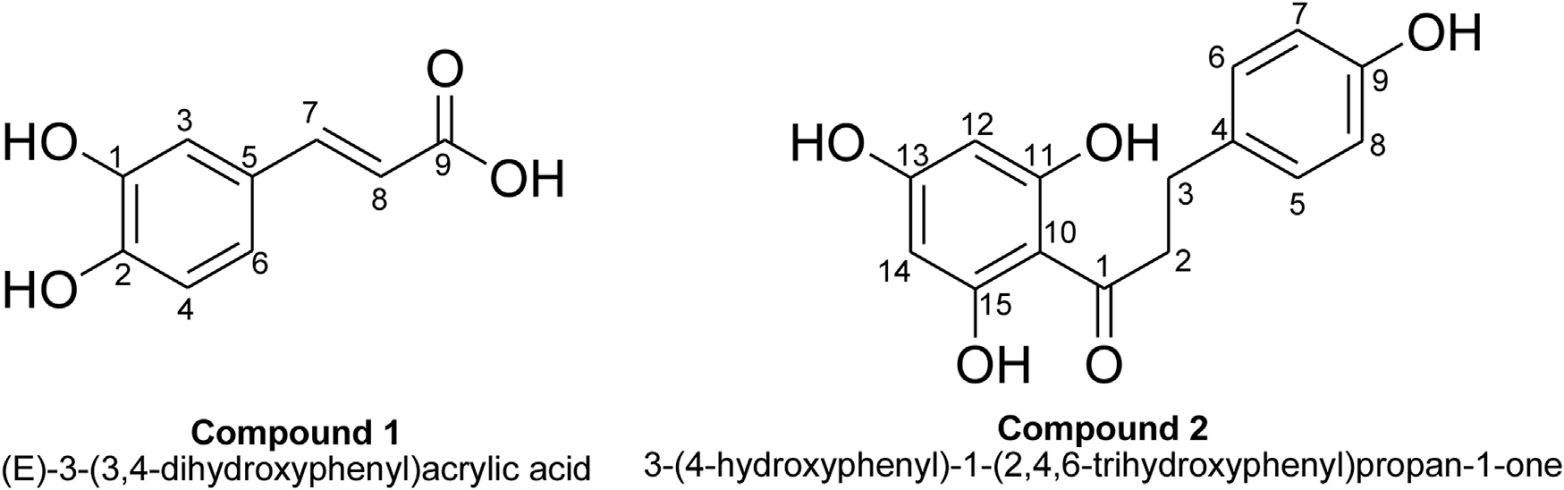
Structures of isolated compounds (1 and 2).

The compounds that were isolated from the chloroform fraction of the plant were purified and identified, as shown in Figure 1.

### 3.2.^1^H and ^13^C NMR spectra of compound 1

^1^H NMR (400 MHz; CD_3_OD), (ppm): 7.52 (d, *J* = 15.91 Hz; 1H; this doublet with a large coupling constant can be allocated to the hydrogen atom at point 7),7.11 (s, 1H; this singlet stands for the hydrogen atom at position 3), 7.06 (d, *J* = 8.29 Hz, 1H; this splitting can be assigned to the hydrogen atom at point 4), 6.88, (d, *J* = 8.30 Hz, 1H; this stands for the hydrogen atom at point 6 on the aromatic ring), 6.26 (d, *J* = 15.91 Hz, 1H; this doublet can be assigned to the hydrogen atom at point 8 due to the large value of the coupling constant indicating trans splitting). ^13^C NMR (100 MHz; CD_3_OD) (ppm): 205.07, 164.49, 155.20, 132.63, 128.96, 114.75, 94.39, 45.92, and 30.15. The ^13^C NMR and ^1^H NMR spectra of compound **1** are displayed in Supplementary Figures S1, S2 in the Supplementary Material. Furthermore, the molecular weight of compound **1** was determined to be 180 using ESI-MS, as displayed in Supplementary Figure S3. The fragmentation pattern of compound **1** by ESI-MS was observed at 180 (100%, molecular ion peak), 159 (17%), 137 (22%), 122 (19%), 94 (15%), 61 (8%), and 43 (18%).

### 3.3 ^1^H and ^13^C NMR spectra of compound 2

^1^H NMR (400 MHz, CD_3_OD) (ppm): 2.75 (t, *J* = 7.56 Hz, 2H; these represent the two hydrogen atoms (-CH2-) next to an aromatic ring, i.e., at positions 2, 3, and 17), (t, *J* = 8.13 Hz, 2H), and 5.71 (s, 2H; singlet associated with hydrogen molecules at positions 12 and 14). As a result, both are indicated as a singlet at chemical shifts of 5.71 and 6.59 (d, *J* = 8.45 Hz, 2H; this doublet may be associated with the two (2) hydrogen atoms at positions 5 and 6) and 6.94 (d, *J*: 8.45 Hz, 2H; these represent the hydrogen molecules at positions 7 and 8). Most likely, the wide singlet at 4.76 represents hydroxyl groups that are chemically comparable.

^13^C NMR (100 MHz; CD_3_OD) (ppm): 169.35, 151.35, 149.38, 145.03, 127.49, 122.46, 115.52, 111.22, 110.10, 55.08, and 54.99. The ^13^C NMR and ^1^H NMR spectra of isolated compound 2 are displayed in Supplementary Figures S4, S5, while the pragmatic molecular weight of isolated compound **2** via ESI-MS was 274, as shown in Supplementary Figure S6. The fragmentation pattern of compound **2** was observed at 274 (100%, molecular ion peak), 242 (10%), 211 (26%), 169 (24%), 141 (18%), 98 (28%), and 67 (36%).

### 3.4 Results of lipoxygenase (5-LOX) inhibitory activity

The 5-LOX inhibitory potential of compounds 1 and 2 confirmed concentration-dependent percent inhibition, as displayed in Table 1. The tested isolated compound **1** confirmed 76.97 ± 1.49, 71.80 ± 0.55, 66.87 ± 1.04, 62.37 ± 0.72, and 59.40 ± 0.65% inhibitions at the concentrations of 1000–62.5 μg/ml accordingly. The IC_50_ value deliberated from the results was noted as 19.59 μg/ml. Similarly, compound **2** did not exhibit brilliant results, showing 41.43 ± 0.38 and 36.72 ± 0.67% inhibition at concentrations of 1000 and 500 μg/ml, respectively. The IC_50_ value measured for zileuton was 3.21 μg/ml.

**TABLE 1:** 5-LOX inhibitory activity of the isolated compounds of *Isodon rugosus*.

Sample	Concentration (µg/ml)	% inhibition 5-LOX (mean ± SEM)	IC_50_ (µg/ml)	IC_50_ (µM)
**Compound 1**	1000	76.97 ± 1.49^***^	19.59	108.74
	500	71.80 ± 0.55^***^		
	250	66.87 ± 1.04^***^		
	125	62.37 ± 0.72^***^		
	62.5	59.40 ± 0.65^***^		
**Compound 2**	1000	41.43 ± 0.38^***^	2573	9381
	500	36.72 ± 0.67^***^		
	250	31.10 ± 0.20^***^		
	125	25.30 ± 0.32^***^		
	62.5	21.11 ± 0.90^***^		
**Zileuton**	1000	95.20 ± 0.15	3.21	13.58
	500	92.22 ± 0.11		
	250	88.98 ± 0.85		
	125	86.20 ± 0.65		
	62.5	79.80 ± 0.37		

Results are shown as the mean ± SEM; two-way ANOVA, followed by Bonferroni’s test, was used; n = 3; ***, *p* < 0.001.

### 3.5 Results of COX-2 activity

The COX-2 inhibitory potential of compounds isolated from *I. rugosus* illustrated concentration-dependent antinociceptive activity. Compound 1 revealed outstanding inhibitory activity with 72.63 ± 0.57, 68.42 ± 0.64, 62.54 ± 0.84, 58.44 ± 0.90, and 51.62 ± 1.20% inhibitions at concentrations ranging from 1000 to 62.5 μg/ml, respectively, with an IC_50_ value of 47.42 μg/ml. Likewise, compound 2 also exhibited moderate activity, causing 52.97 inhibitions at the highest concentration. Similarly, for celecoxib, the IC_50_ value calculated was noted as 1.41 μg/ml, as shown in Table 2.

**TABLE 2:** COX-2 percent inhibitory activity of isolated compounds from *Isodon rugosus*.

Sample	Concentration (µg/ml)	% inhibition, COX-2 (mean ± SEM)	IC_50_ (µg/ml)	IC_50_ (µM)
**Compound 1**	1000	72.63 ± 0.57^***^	47.98	266.32
	500	68.42 ± 0.64^***^		
	250	62.54 ± 0.84^***^		
	125	58.44 ± 0.90^***^		
	62.5	51.62 ± 1.20^***^		
**Compound 2**	1000	52.97 ± 0.79^***^	790.13	2880
	500	45.10 ± 0.22^***^		
	250	41.10 ± 0.24^***^		
	125	36.33 ± 0.37^***^		
	62.5	31.90 ± 0.92^***^		
**Celecoxib**	1000	97.55 ± 0.40	1.41	3.70
	500	92.37 ± 1.65		
	250	90.50 ± 0.40		
	125	88.60 ± 0.90		
	62.5	85.17 ± 0.72		

Results are shown as the mean ± SEM; two-way ANOVA, followed by Bonferroni’s test, was used; all the values were significantly different from those of the positive control; n = 3; ***, *p* < 0.001.

### 3.6 Acute toxicity

When conducting the acute toxicity test, there was no mortality or behavioral changes observed at the designated doses. Therefore, a dosage of up to 2000 mg/kg was deemed safe for the test compounds.

### 3.7 Result of the writhing test

The analgesic effect of compounds 1 and 2 through an acetic acid-induced writhing test demonstrated significant activity. The positive control exhibited a mean writhing inhibition of 16.5 ± 0.76 at the 10 mg/kg dosage, resulting in a 73.02% inhibition. Compound 1 showed 05.10, 19.88, 24.78, and 61.90% inhibition at doses of 10, 20, 25, and 50 mg/kg, respectively, with mean writhes of 58.00 ± 1.15, 49.33 ± 0.87, 46.67 ± 0.87, and 23.30 ± 0.62. However, compound 2 revealed a mean writhing inhibition of 60.02 ± 0.90 with 01.86% inhibition at the highest dose of 10 mg/kg, as evident from Table 3.

**TABLE 3:** Acetic acid-induced writhing results of the isolated compounds.

Sample	Dose (mg/kg)	Mean writhes	% analgesic activity
**Negative control**	---	61.16 ± 0.74	0.00
**Compound 1**	10	58.00 ± 1.15	05.10^ns^
	20	49.33 ± 0.87	19.88^ns^
	25	46.67 ± 0.87	24.78^**^
	50	23.30 ± 0.62	61.90^***^
**Compound 2**	10	60.02 ± 0.90	1.86^ns^
	20	55.35 ± 0.87	9.49^ns^
	25	49.90 ± 0.20	18.41^*^
	50	41.60 ± 1.24	31.98^***^
**Positive control**	10	16.5 ± 0.76	73.02^***^

Results are shown as mean ± SEM; two-way ANOVA, followed by Bonferroni’s test, was used; all the values were significantly different from those of the positive control; n = 5; ***, *p* < 0.001, **, *p* < 0.01, and *, *p* < 0.05, ns; non-significant.

### 3.8 Hot plate test

During the hot plate test, compound 1 effectively improved the reaction time, displaying 08.22 ± 0.07, 07.19 ± 0.08, 6.65 ± 0.05, 8.38 ± 0.41, and 7.17 ± 0.57 from 15 to 90 min, correspondingly (Table 4). In the early 15 min, compound 1, when administered with naloxone, exhibited a reaction time recorded as 05.21 ± 0.06 s. Similarly, preceding 90 min, the compound with naloxone showed a reaction time documented as 4.20 ± 0.48 s Compound 1 was considerably more active, while compound 2 displayed minimal analgesic potential.

**TABLE 4:** Analgesic effect following the hot plate model and opioid receptor evaluation study.

Sample	Dose (mg/kg)	Reaction time on the hot plate (minutes)				
		15	30	45	60	90
**Negative control**	---	03.72 ± 0.42	04.37 ± 0.57	2.95 ± 0.33	2.93 ± 0.50	2.70 ± 0.44
**Mor**	5	11.16 ± 0.94^###^	10.46 ± 0.66^###^	9.73 ± 0.88^###^	9.73 ± 0.60^###^	7.36 ± 0.94^###^
**Mor + Nal**	5 + 1	04.76 ± 0.30^***^	05.96 ± 0.54^***^	3.96 ± 0.40^***^	3.60 ± 0.76^***^	2.90 ± 0.36^***^
**Compound 1**	50	08.22 ± 0.07^*^	07.19 ± 0.08^**^	6.65 ± 0.05^***^	8.38 ± 0.41^*^	7.17 ± 0.57^**^
**Compound 2**	50	03.84 ± 0.03^***^	04.52 ± 0.09^***^	3.27 ± 0.48^***^	3.18 ± 0.34^***^	2.85 ± 0.29^***^
**Compound 1 + Nal**	50 + 1	05.21 ± 0.06^***^	06.22 ± 0.05^***^	4.39 ± 0.55^***^	3.97 ± 0.30^***^	4.20 ± 0.48^***^

Results are shown as mean ± SEM; two-way ANOVA, followed by Bonferroni’s test, was used; all the values were significantly different from those of the positive control; n = 5; ***, *p* < 0.001, **, *p* < 0.01, and *, *p* < 0.05; ns, non-significant compared to that of the positive control; = *p* < 0.001 comparison of the positive control to the negative control.

### 3.9 Opioid receptor involvement

The results shown by compound 1 in the hot plate test exhibited the same effect as morphine. The effects of compound 1 were efficiently eliminated by an opioid antagonist (naloxone). The reaction time in the hot plate test was significantly reduced by administering naloxone, which verified the association of opioid receptors in the analgesic corridor of compound 1.

### 3.10 Formalin test result and opioid receptor involvement

The formalin test was used to evaluate the antinociceptive assay using mouse models by injecting 2% of formalin (intraplantar) into the mice, which produced the characteristic bi-phasic licking reaction. The time interval of licking for the first phase (0–5 min) was measured as 56.64 ± 0.76 s, and for the second phase (15–30 min), it was 78.78 ± 0.70 s in the control group. The administration of morphine at a dose of 5 mg/kg intraperitoneally displayed noticeable potential in alleviating neurogenic pain. During the formalin test, pain caused by inflammation was inhibited in the initial stage (84.99%) and final stage (91.33%). The initial stage activity of morphine plus naloxone was 7.61%, while the final stage activity was 14.37%. Compound 1 showed 69.45% inhibition in the first stage and 63.85% in the second phase. Compound 2 was found inactive in both phases. Naloxone significantly reversed the antinociceptive effect of compound 1, similar to that of morphine. Naloxone dramatically inhibited the analgesic action of compound 1 in both the early neurogenic stage and the late pathogenic phase, indicating that the chemical is involved in the opioidergic cascade of pain regulation, as shown in Table 5.

**TABLE 5:** Effect of compound 1 on formalin-induced pain in mice.

Sample	Dose (mg/kg)	Total time spent in licking			
		0–5 min	% inhibition	15–30 min	% inhibition
**Negative control**	---	56.64 ± 0.76	---	78.78 ± 0.70	---
**Mor**	5	08.50 ± 0.76	84.99^###^	06.83 ± 0.60	91.33^###^
**Mor + Nal**	5 + 1	52.33 ± 0.66	7.61^**^	67.50 ± 0.42	14.37^***^
**Compound 1**	50	17.30 ± 0.77	69.45^***^	28.48 ± 0.58	63.97^***^
**Compound 2**	50	51.55 ± 0.99	8.84^**^	67.83 ± 0.57	13.99^***^
**Compound 1 + Nal**	50 + 1	43.35 ± 0.52	23.36^***^	51.20 ± 0.94	35.17^***^

Data were shown as mean ± SEM; two-way ANOVA, followed by Bonferroni’s test, was used; all the values were significantly different from those of the positive control; n = 5; ***, *p* < 0.001, **, *p* < 0.01, and *, *p* < 0.05; ns, non-significant; Mor, morphine; Nal, naloxone, compared to that of the positive control; = *p* < 0.001 comparison of the positive control to the negative control.

### 3.11 Adrenergic receptor involvement

The antinociceptive activity of compounds 1 and 2 was assessed, with compound 1 exhibiting significant activity, while compound 2 showed weak analgesic activity, as shown in Table 6. Pre-administering yohimbine (an alpha-2 antagonist) to mice has the ability to reverse the action of alpha-2 receptor agonist (clonidine). Clonidine showed reaction times of 10.12 ± 0.67, 9.77 ± 0.75, 10.30 ± 0.50, 8.60 ± 0.78, and 9.32 ± 0.68 min at the interval of 15–90 min accordingly. Although pre-administration of yohimbine to test models reversed the activity of clonidine and revealed the action in the existence of yohimbine, the reaction time was measured as 3.87 ± 0.65, 4.47 ± 0.54, 3.83 ± 0.50, 3.65 ± 0.44, and 2.95 ± 0.29 min at the periods of 90, 60, 45, 30, and 15 min, respectively. Similarly, compound 1 demonstrated reaction times of 5.43 ± 0.52, 5.21 ± 0.45, 6.65 ± 0.74, 5.89 ± 0.63, and 4.75 ± 0.41 min in the same time interval. The pre-administration of yohimbine reversed the activity of compound 1, measured as 3.61 ± 0.53, 4.32 ± 0.52, 4.88 ± 0.44, 3.52 ± 0.54, and 3.36 ± 0.56 min at the intervals of 15–90 min, respectively, which confirmed the connection of adrenergic receptors in the intonation of pain. The current trial exposed the function of adrenergic receptors in the inflection of pain.

**TABLE 6:** Result of analgesic activity following the adrenergic pathway.

Sample	Dose (mg/kg)	Reaction time on the hot plate				
		15 min	30 min	45 min	60 min	90 min
**Negative control**	---	2.40 ± 0.26	3.28 ± 0.44	3.57 ± 0.47	3.27 ± 0.48	2.97 ± 0.43
**Compound 1**	50	5.43 ± 0.52^***^	5.21 ± 0.45^***^	6.65 ± 0.74^***^	5.89 ± 0.63^**^	4.75 ± 0.41^***^
**Compound 2**	50	2.57 ± 0.21^ns^	3.42 ± 0.46^ns^	3.58 ± 0.40^ns^	3.37 ± 0.48^ns^	3.15 ± 0.45^*^
**Clonidine**	3 μg/kg	10.12 ± 0.67^***^	9.77 ± 0.75^***^	10.30 ± 0.50^***^	8.60 ± 0.78^***^	9.32 ± 0.68^***^
**Clonidine + yohimbine**	3 μg/kg + 1 mg/kg	2.95 ± 0.29^*^	3.65 ± 0.44^ns^	3.83 ± 0.50^ns^	4.47 ± 0.54^*^	3.87 ± 0.65^ns^
**Compound 1 + yohimbine**	50 + 1 mg/kg	3.61 ± 0.53^**^	4.32 ± 0.52^*^	4.88 ± 0.44^*^	3.52 ± 0.54^ns^	3.36 ± 0.56^ns^

Results are shown as mean ± SEM; two-way ANOVA, followed by Bonferroni’s test, was used; all the values were significantly different from those of the positive control; n = 5; ***, *p* < 0.001, **, *p* < 0.01, and *, *p* < 0.05; ns, non-significant; Mor, Morphine; Nal, naloxone.

### 3.12 Evaluation of anti-inflammatory activity

**FIGURE 2:**
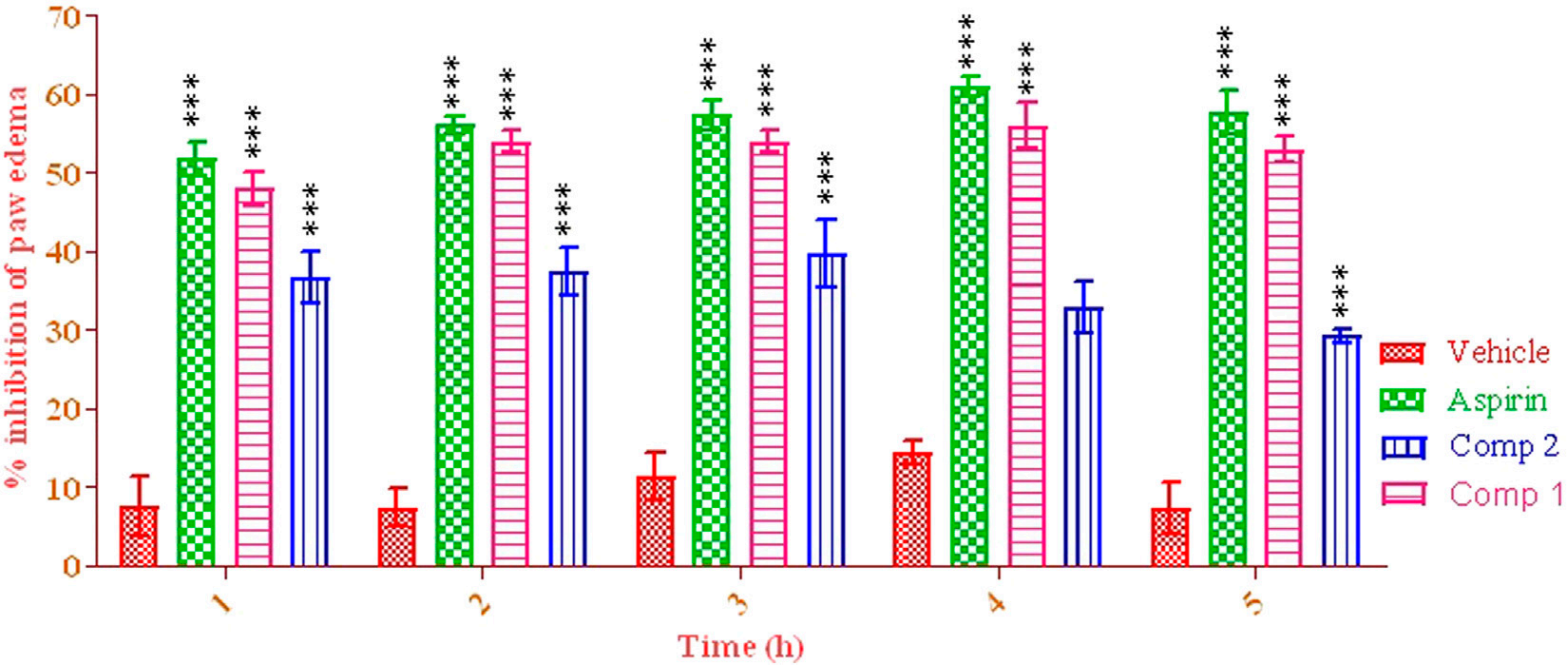
Anti-inflammatory potential of pure isolated compounds. Two-way ANOVA was used, followed by Bonferroni’s posttest. Data were analyzed in triplicate (mean ± SEM). ^***^ *p* < 0.001.

The mice administered with aspirin 10 mg/kg showed a considerable reduction in paw volume compared to the vehicle control group. The positive control group showed a reduction in the percentage increase of paw volume from the initial 1st to the 5th hour, ranging from 51.77 ± 0.10 to 57.77 ± 0.40 (*p* < 0.001), indicating % edema volume inhibition. Compound 1 displayed the highest activity at 4th h (56.40 ± 0.42) after carrageenan induction. Compound 2 was not effective against carrageenan-induced inflammation, causing the highest percent inhibition at the first hour (35.10 ± 0.22%), as shown in Figure 2.

### 3.13. SwissADME studies of the isolated compounds

**FIGURE 3:**
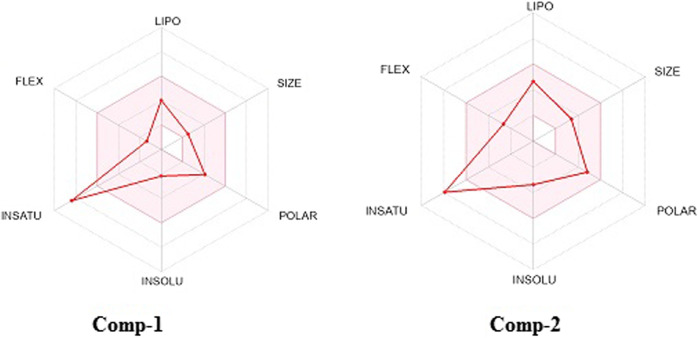
Physicochemical properties radar chart of isolated compounds.

The physicochemical properties of isolated compounds are displayed in Table 7 and Figure 3. According to the table, the physicochemical properties of compounds 1 and 2 were examined and alienated into the six (6) main groups and also studied for suitable ranges for oral bioavailability. Molecular weight, topological polar surface area (TPSA), no. of H-bond donors, LogP, and number of hydrogen bond acceptors (NHBAs) of the isolated compounds were within documented limits of ≤500, ≤5, ≤140 A^2^, and ≤10, respectively. F. Csp^3^ values range from 0.10 to 0.13. Compounds 1 and 2 have 2 and 4 rotatable bonds, respectively. The molar refractivity range is between 52.12 and 74.02.

**TABLE 7:** Pharmacokinetic studies of the isolated compounds calculated using the SwissADME database.

Physicochemical property		
	Compound 1	Compound 2
**Formula**	C_10_H_10_O_4_	C_15_H_14_O_5_
**Molecular weight**	194.18 g/mol	274.27 g/mol
**Heavy atoms**	14	20
**Fraction Csp3**	0.10	0.13
**Number of aromatic heavy atoms**	6	12
**Number of rotatable bonds**	2	4
**Number of H-bond acceptors**	4	5
**Number of H-bond donors**	3	4
**Molar refractivity**	52.12	74.02
**TPSA**	77.76 Å^2^	97.99 Å^2^
SMILES notation		
	CC1 = C (\C=C\C(O) = O)C=CC(O) = C1O	Oc1ccc (cc1)CCC(=O)c1c(O)cc (cc1O)O
Lipophilicity		
**Consensus Log *P***_**o/w**_	1.26	1.93
**Log *P***_**o/w**_ **(SILICOS-IT)**	1.25	2.17
**Log *P***_**o/w**_ **(MLOGP)**	1.00	1.10
**Log *P***_**o/w**_ **(WLOGP)**	1.40	2.32
**Log *P***_**o/w**_ **(XLOGP3)**	1.53	2.63
**Log *P***_**o/w**_ **(iLOGP)**	1.13	1.41
Water solubility		
**Class**	Soluble	Soluble
**Solubility**	1.54e^+01^ mg/ml; 7.91e^-02^ mol/L	1.16e^-01^ mg/ml; 4.25e^-04^ mol/L
**Log *S* (SILICOS-IT)**	-1.10	-3.37
**Class**	Soluble	Moderately soluble
**Solubility**	3.28e^01^ mg/ml; 1.69e^-03^ mol/L	1.26e^-02^ mg/ml; 4.59e^-05^ mol/L
**Log *S* (Ali)**	-2.77	-4.34
**Solubility**	1.25e^+00^ mg/ml; 6.41e^-03^ mol/L	1.15e^-01^ mg/ml; 4.19e^-04^ mol/L
**Log *S* (ESOL)**	-2.19	-3.38
Pharmacokinetics		
**Log *K***_**p**_ **(skin permeation)**	-6.40 cm/s	-6.11 cm/s
**CYP3A4 inhibitor**	No	Yes
**CYP2D6 inhibitor**	No	No
**CYP2C9 inhibitor**	No	Yes
**CYP2C19 inhibitor**	No	No
**CYP1A2 inhibitor**	No	Yes
**P-gp substrate**	No	No
**BBB permeant**	No	No
**GI absorption**	High	High
Druglikeness		
**Bioavailability score**	0.56	0.55
**Muegge**	No; 1 violation: MW < 200	Yes
**Egan**	Yes	Yes
**Veber**	Yes	Yes
**Ghose**	Yes	Yes
**Lipinski**	Yes; 0 violation	Yes; 0 violation
Medicinal chemistry		
**Synthetic accessibility**	2.00	1.88
**Lead-likeness**	No; 1 violation: MW < 250	Yes
**Brenk**	2 alerts: catechol and michael_acceptor_1	0 alert
**PAINS**	1 alert: catechol_A	0 alert

**FIGURE 4:**
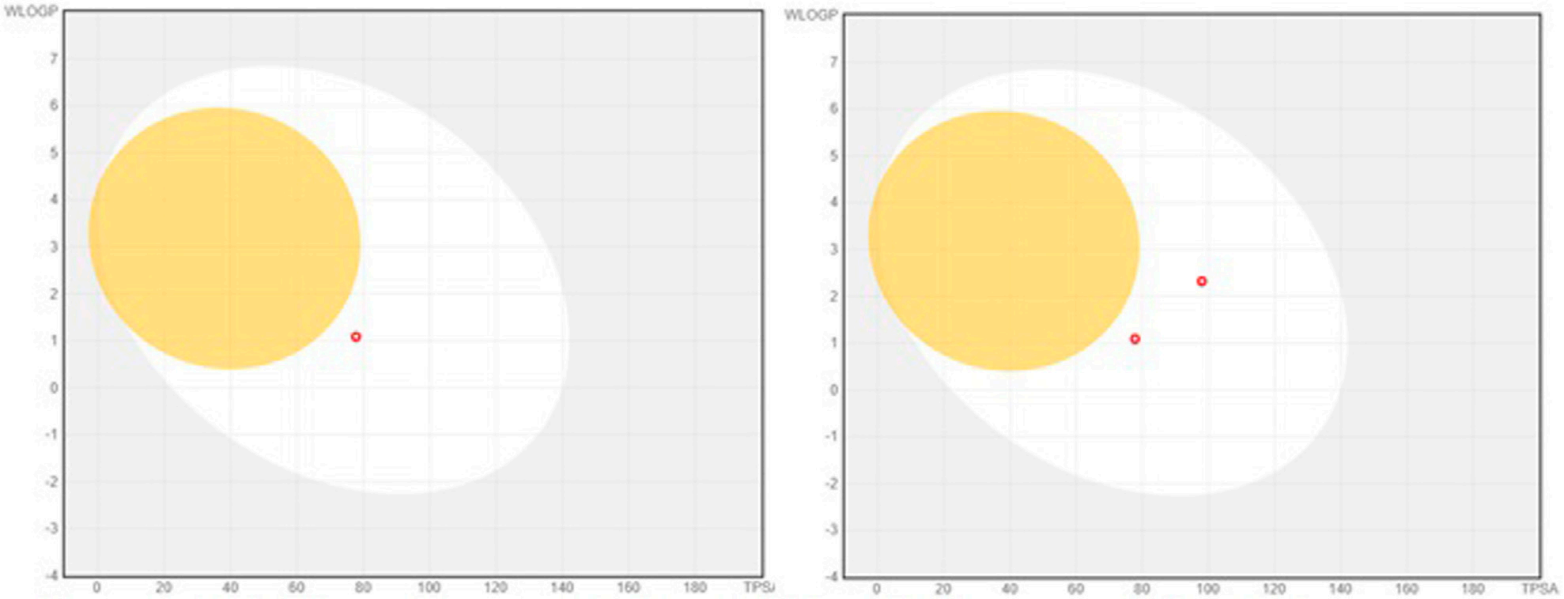
Boiled graph representation of the isolated compounds 1 and 2.

The pharmacokinetic properties of molecules are very significant for achieving the desired pharmacological effect for a drug candidate. This suggests that each pharmacokinetic factor might impact the biological characteristics of molecules. The SwissADME database exposed high GI absorption for the isolated compounds. The graph of the boiled egg of the isolated compounds is displayed in Figure 4 accordingly. Both compounds showed high absorption of GI. The compounds are unable to cross the blood–brain barrier. Compound 2 was known as an inhibitor of CYP1A2, CYP3A4, and CYP2C9 in drug metabolism. Compound 1 has no effect on CYP1A2, CYP2C19, CYP2C9, CYP2D6, and CYP3A4. Similarly, the isolated compounds have drug-likeness parameters, i.e., Muegge, Lipinski, Veber, Ghose, and Egan rules of drug likeness. Compound 1 had just one infraction of the Muegge rule (MW < 200). The bioavailability scores for compounds 1 and 2 were 0.56 and 0.55, respectively. The isolated compounds have no PAINS alert for compound 2, while compound 1 showed one alert for catechol_A, free from *α*-screen artifacts, reactive compounds, and frequent hitters. Two reactive groups in compound 1 have been identified through Brenk structural alerts, namely, catechol and Michael_acceptor_1. Compound 1 has MW < 250, resulting in likeness capability. Compound 2 lacks the ability to lead in terms of likeness. The SwissADME database has assigned scores ranging from 2.00 to 1.88 for synthetic accessibility.

## 4 Discussion

The goal of the current research work is to investigate the antinociceptive effects of pure compounds isolated from *I. rugosus* to confirm and verify the traditional utilization of this plant. Experimental animal models were considered for conducting this research, and various assays have been carried out, including the hot plate test, acetic acid-induced writhing, formalin-induced paw licking assay, adrenergic receptor involvement, and lipoxygenase and cyclooxygenase inhibitory assay. The current project has been carried out based on the results of crude samples in a previous study (Zeb et al., 2016; Mahnashi et al., 2022a). Generally, various types of models are employed for the evaluation of the analgesic effects of test drugs. The most widely employed models use thermal and chemical nociception models. The hot plate test uses thermal nociception and chemical nociception, which are employed in the investigational models of formalin- and acetic acid-induced tests. Previous research has shown that the hot plate experiment uses the central corridor for pain modulation, whereas the peripheral system is involved in acetic acid-induced writhing. The formalin assay is also considered to show the participation of both peripheral and central routes (Hunskaar and Hole, 1987). Additionally, it is suggested that acetic acid could contribute to the production of naturally occurring mediators such as prostaglandin E and lipoxygenase products in the intraperitoneal liquids, which results in activating the nearby nociceptive receptors. In the same way, the adrenergic receptors are responsible for pain modulation, i.e., the activation of these receptors has been reported to modulate the pain signals (Owoyele et al., 2013; Mahnashi et al., 2022b). The current research work generally puts forward the role of peripheral and central and enzymatic pathways in the suppression of pain. Compound 1 is phenolic acid, 3,4-dihydroxy-cinnamic acid containing acrylic and phenolic functionalities, which have been previously found in various aromatic herbs like sage, rosemary, spearmint, thyme, lavender, and balm (Wang et al., 2004). Compound 1 has been confirmed for its anti-coagulatory, analgesic, and anti-inflammatory potentials (Chao et al., 2009). Da Cunha et al. (2004) synthesized five derivatives of caffeic acid, namely, benzyl, octyl, butyl, ethyl, and methyl, which were explored for their anti-inflammatory potential. Mehrotra et al. (2011) evaluated the antinociceptive profile of commercially available caffeic acid in mice and rats.

Compound 1 is also called 2-4-4-6-tetrahydroxy-dihydrochalcone (dihydronaringenin) (Rezk et al., 2002). It is found in pears that have been checked for their anti-tumor activity via inhibiting the protein kinase (Kern et al., 2007). Al-Ostoot et al. (2021) synthesized caffeic acid and explored for its anti-inflammatory, analgesic, and ulcerogenic effects (Al-Ostoot et al., 2021). Similarly, phloretin is a dihydrochalcone flavonoid present in apples with various pharmacological activities like anti-tumor, anti-diabetic, and antioxidant activities. M Balaha explored phloretin along with duloxetin for STZ-induced diabetic neuropathy in rats (Balaha et al., 2018). The effect of caffeic acid and phloretin has not been much envisaged in correlating their effect on inflammation-associated pain. Therefore, the current study was designed to isolate and evaluate the effect of these compounds on different anti-inflammatory and nociceptive paradigms.

As per the results of current investigations, compound 2 has negligible analgesic and anti-inflammatory activities in almost all the test models. As far as compound 1 is concerned, the results of compound 1 in almost all the analgesic and anti-inflammatory models are comparable with the effect of the positive control. Compound 1 was significantly implicated in anti-nociception via opioid receptors, according to the mechanistic investigation. After pre-administration of the opioid blocker naloxone, the mice displayed morphine-like behavior, indicating that the central route via the opioidergic receptor may be the mechanism of action. The two models clearly show that opioid receptors are involved, i.e., the hot plate and formalin, where naloxone was unemployed to sort out the participation of opioid receptors. Likewise, compound 1 has also been established to be involved in the modulation of pain via the dopaminergic pathway (Alqahtani et al., 2022).

The pharmacokinetic evaluation of the isolated compounds exhibited that Lipinski’s rule of five is essential for lucid drug design. Any compound that breaks just one of the requirements might have inadequate absorption or limited permeability (Pathak et al., 2017). Fsp3 is the portion of sp3 carbon atoms out of the entire amount of carbon. This illustrates the intricacy of the molecular arrangement and represents the carbon concentration. An optimal value for Fsp3 is deemed to be ≥0.42, given that approximately 84% of approved drugs fulfill these criteria (Kombo et al., 2013). The H-bond acceptor and donor parameters are maintained within precise and stringent limits. Oral drugs typically show a lower count of H-bond acceptors, donors, and rotatable bonds (Lipinski, 2004; Ahmad et al., 2021b). These three criteria favor the suitability of the oral route of administration due to its flexibility, convenience, and simplicity.

Molecules possessing a TPSA exceeding 140 Å^2^ would exhibit limited absorption, resulting in fractional absorption lower than 10%. Conversely, those with a TPSA of 60 Å^2^ would experience efficient absorption, leading to a fractional absorption of more than 90% (Clark, 1999). The isolated compounds are anticipated to exhibit improved absorption, an inference drawn from their TPSA values (Mahmood et al., 2022). Furthermore, Log Po/w, an amalgamation of iLOGP, XLOGP3, WLOGP, MLOGP, and SILICOS-IT, as calculated by SwissADME, represents the partition coefficient between octanol and water. A higher Log Po/w value indicates increased lipophilicity, influenced by factors such as polarity, molecular size, and hydrogen bonding. The Log Po/w values of both compounds reflect their partition, preferably into the water compartment. However, values of log Po/w show optimal lipophilicity (optimal: 0 < log *p* < 3) (Bitew et al., 2021). These values are somehow congruent with log S values. The SwissADME database revealed high GI absorption (Daina et al., 2017). Most drugs and endogenous chemicals are metabolized by the CYP3A4 system. The bioavailability score indicates an excellent enough plasma concentration. The calculation of bioavailability and permeability is important before proceeding with any advanced testing. Therefore, a probability-based score is given to a drug candidate to have F > 10% (Martin, 2005).

The compounds adhere to several well-recognized rules for drug-likeness, including Lipinski, Muegge, Ghose, Veber, and Egan criteria. Moreover, the absorption of the isolated compounds was found to be safe and is known as the boiled-egg graphical representation. Compounds 1 and 2 showed good GI absorption. None of the compounds exhibit blood–brain barrier permeability, suggesting a favorable characteristic of being devoid of CNS toxicity.

## 5 Conclusion

The current study confirms that *Isodon rugosus* has great value as a source of various compounds for medicinal applications. A potent COX-2 and 5-LOX activity was detected for compound 1 (caffeic acid). Moreover, compound 1 exhibited anti-inflammatory and analgesic activities in animal models of carrageenan-induced inflammation, the hot plate test, and the writhing test. The pharmacokinetic profiles of the isolated compounds were also explored in this research. These pharmacological activities may be due to the inhibitory potential of the synthesis and/or release of various mediators like PGE_2_, leukotrienes, arachidonic acid, and TNF-α. Additional exploration of *I. rugosus* may lead to the promotion and development of novel analgesic and anti-inflammatory compounds.

## Data Availability

The original contributions presented in the study are included in the article/Supplementary Material; further inquiries can be directed to the corresponding authors.
