# Supplementary figures and images for: Investigation of anti-nociceptive, anti-inflammatory potential and ADMET studies of pure compounds isolated from Isodon rugosus Wall. ex Benth
Source: Front Pharmacol. 2024 Feb 13;15:1328128. doi: 10.3389/fphar.2024.1328128 (PMC10897015; doi:10.3389/fphar.2024.1328128)

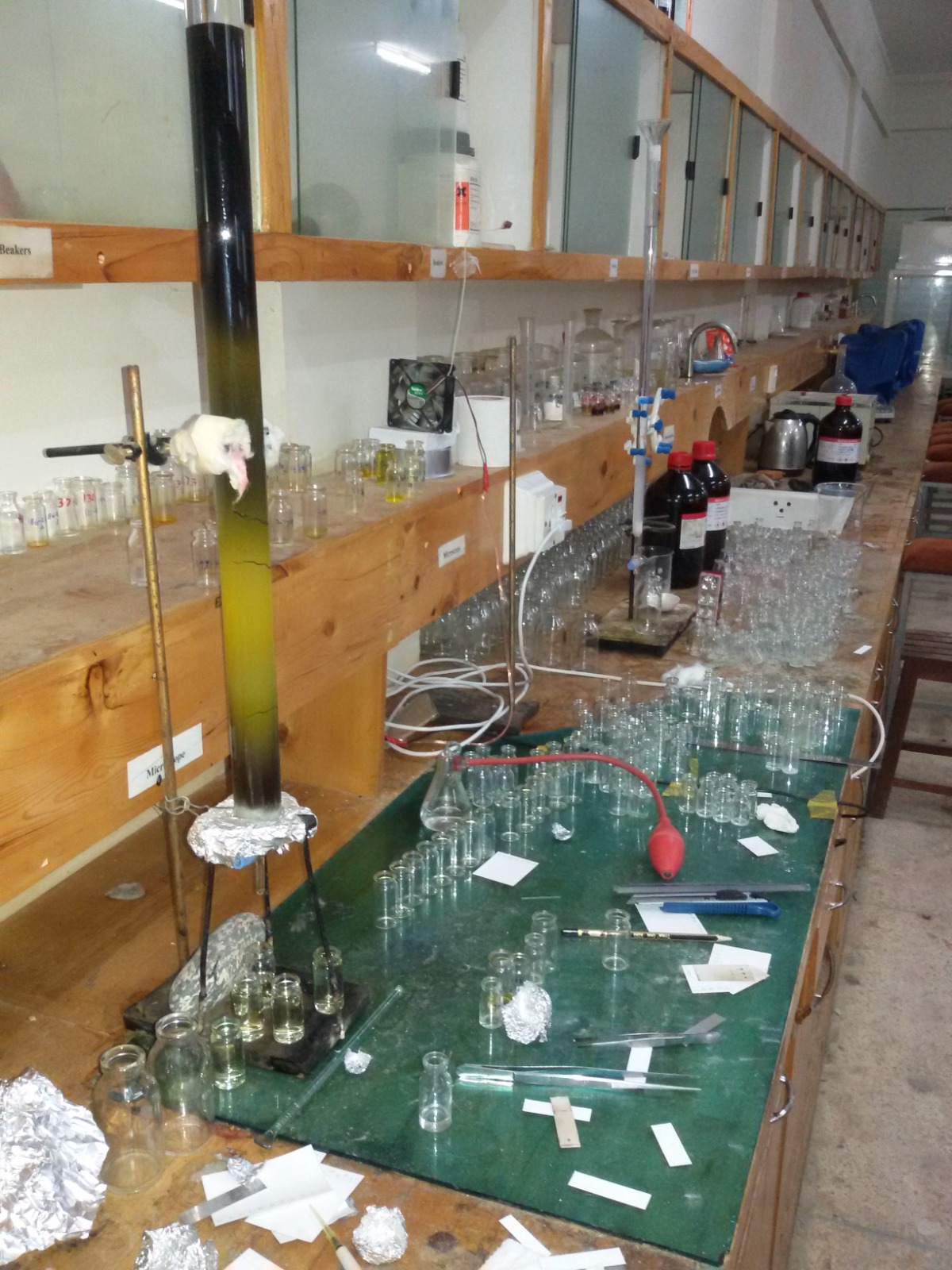

Supplement: Supplementary file 1 [file Image3.JPEG]

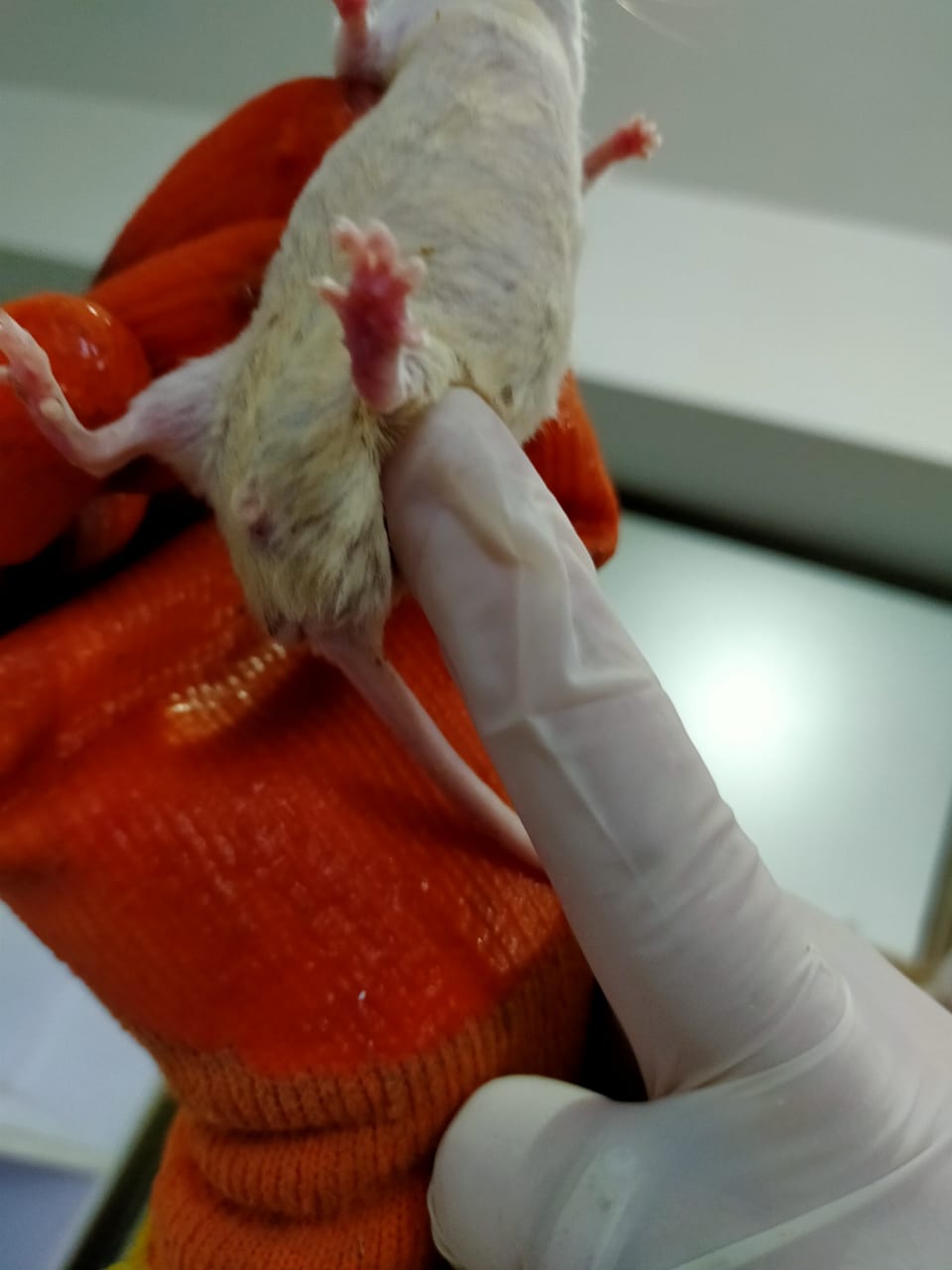

Supplement: Supplementary file 4 [file Image9.JPEG]

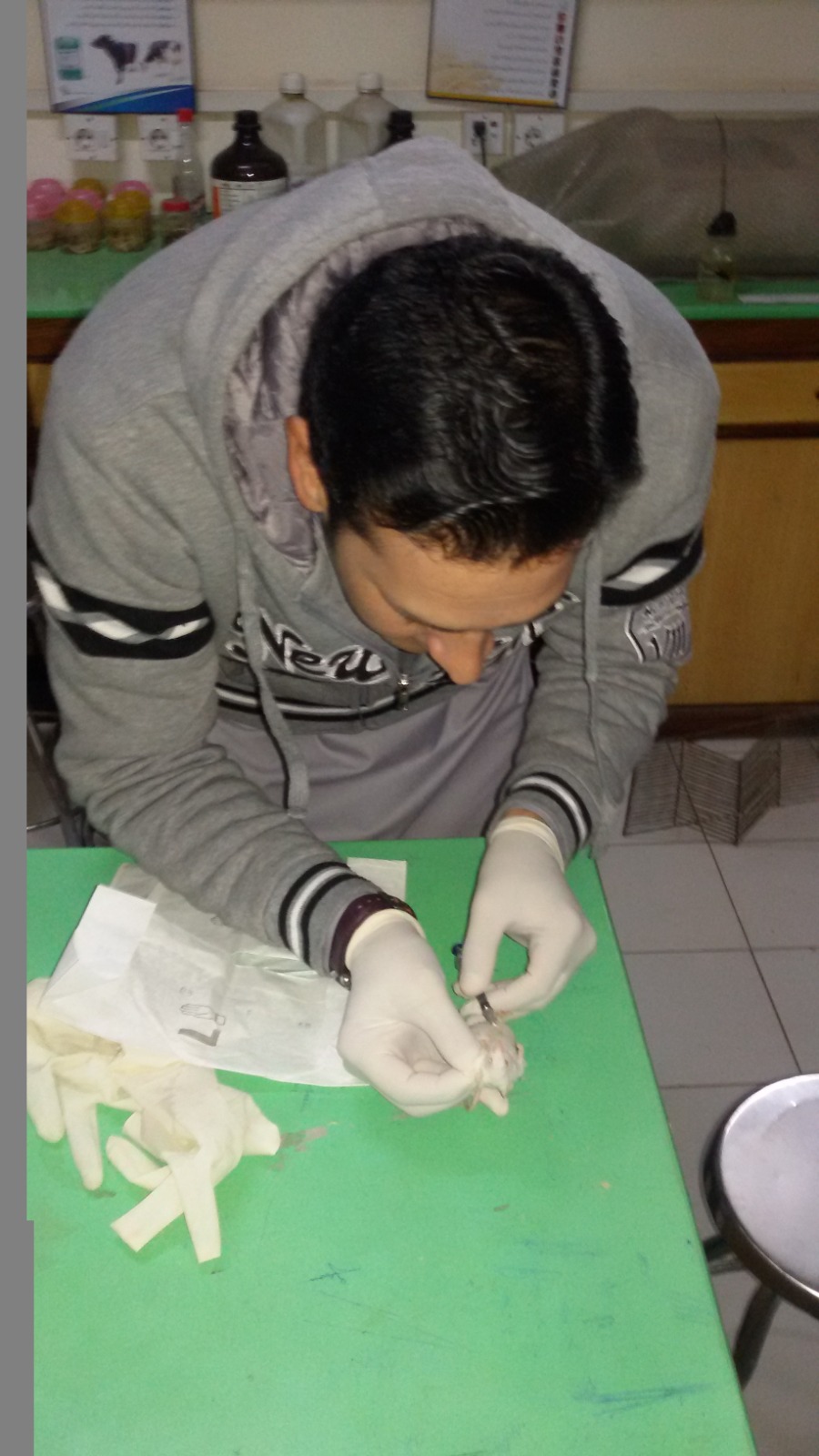

Supplement: Supplementary file 5 [file Image1.JPEG]

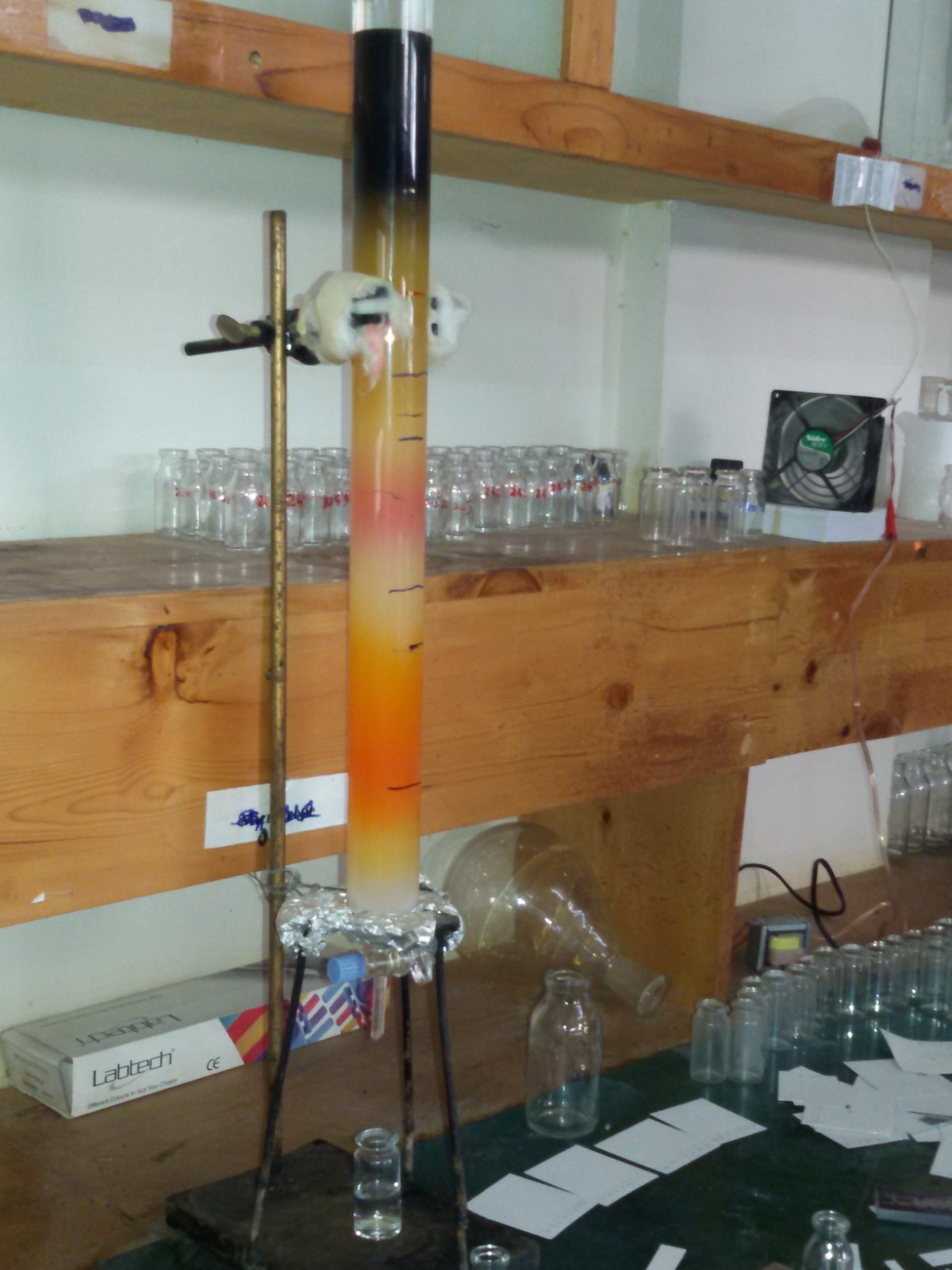

Supplement: Supplementary file 6 [file Image4.JPEG]

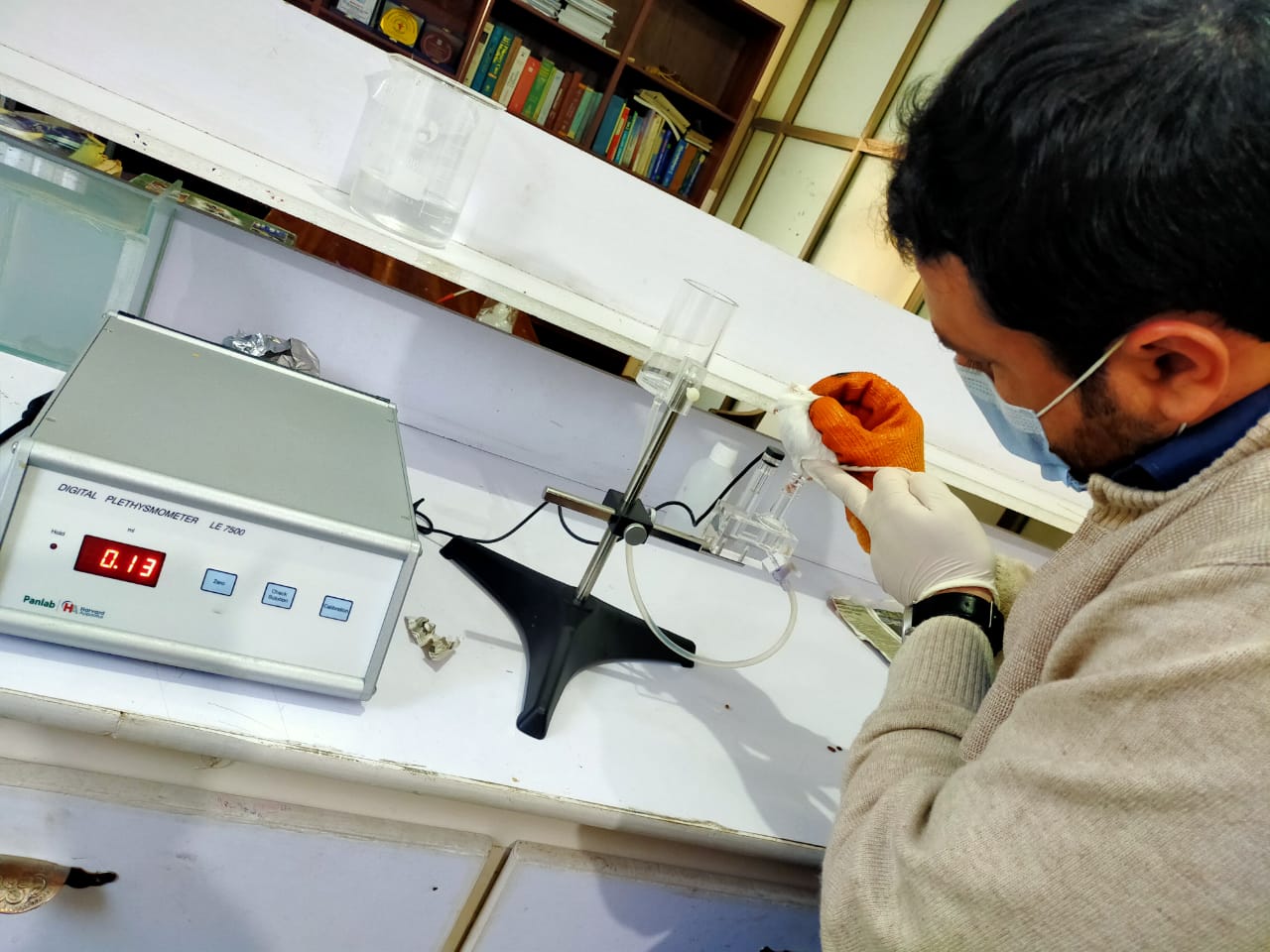

Supplement: Supplementary file 7 [file Image7.JPEG]

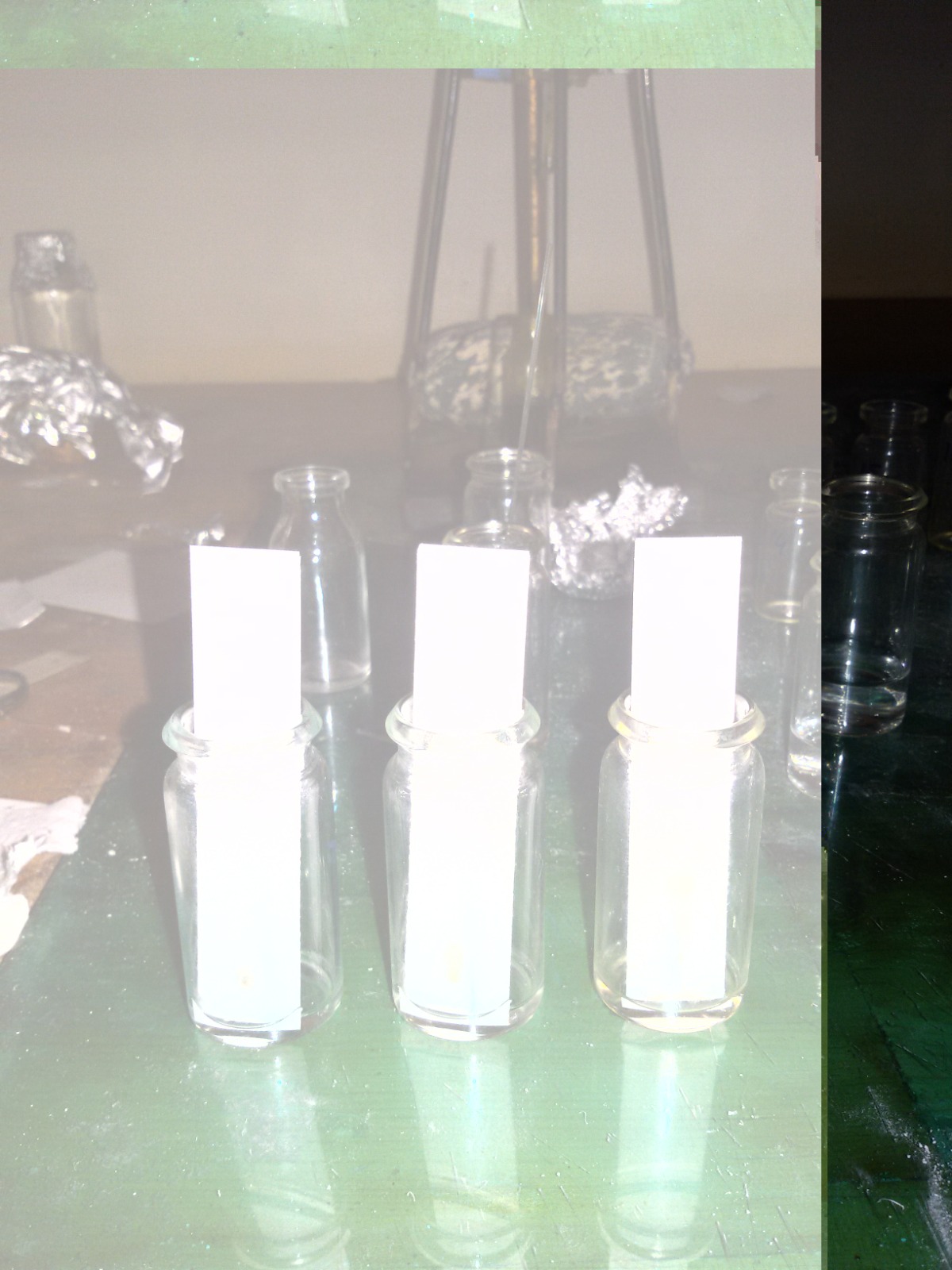

Supplement: Supplementary file 8 [file Image2.JPEG]

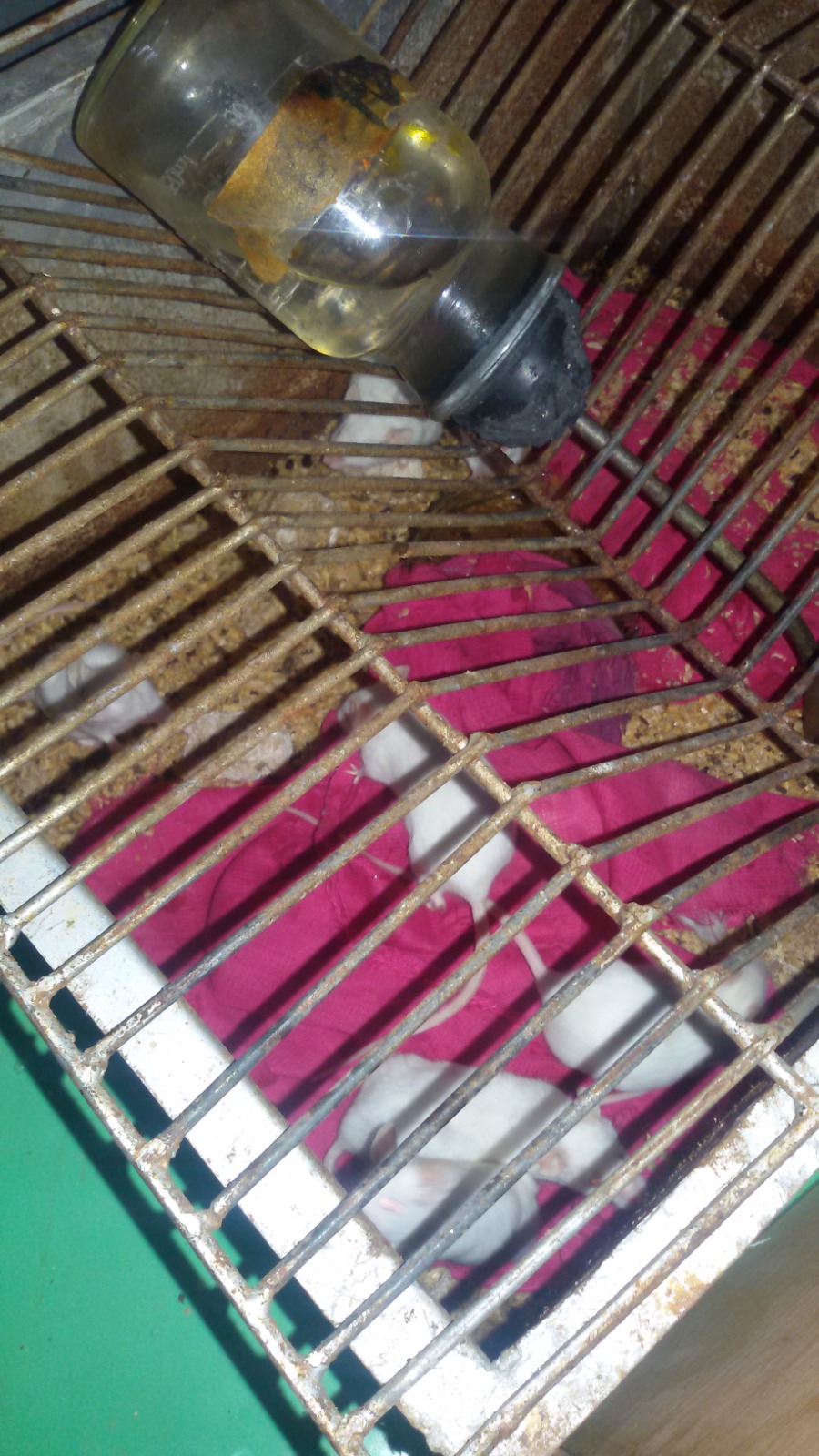

Supplement: Supplementary file 9 [file Image5.JPEG]

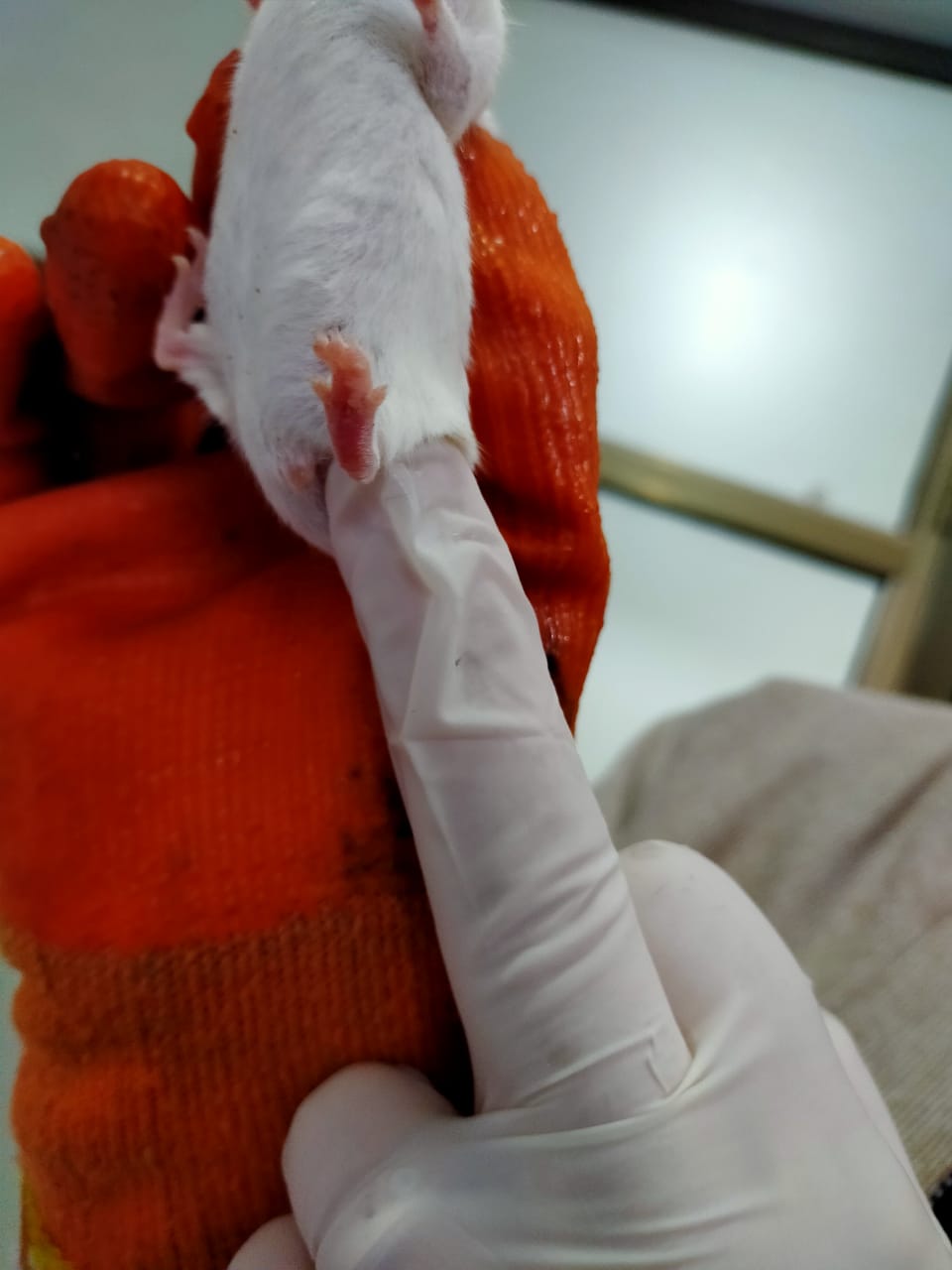

Supplement: Supplementary file 10 [file Image10.JPEG]

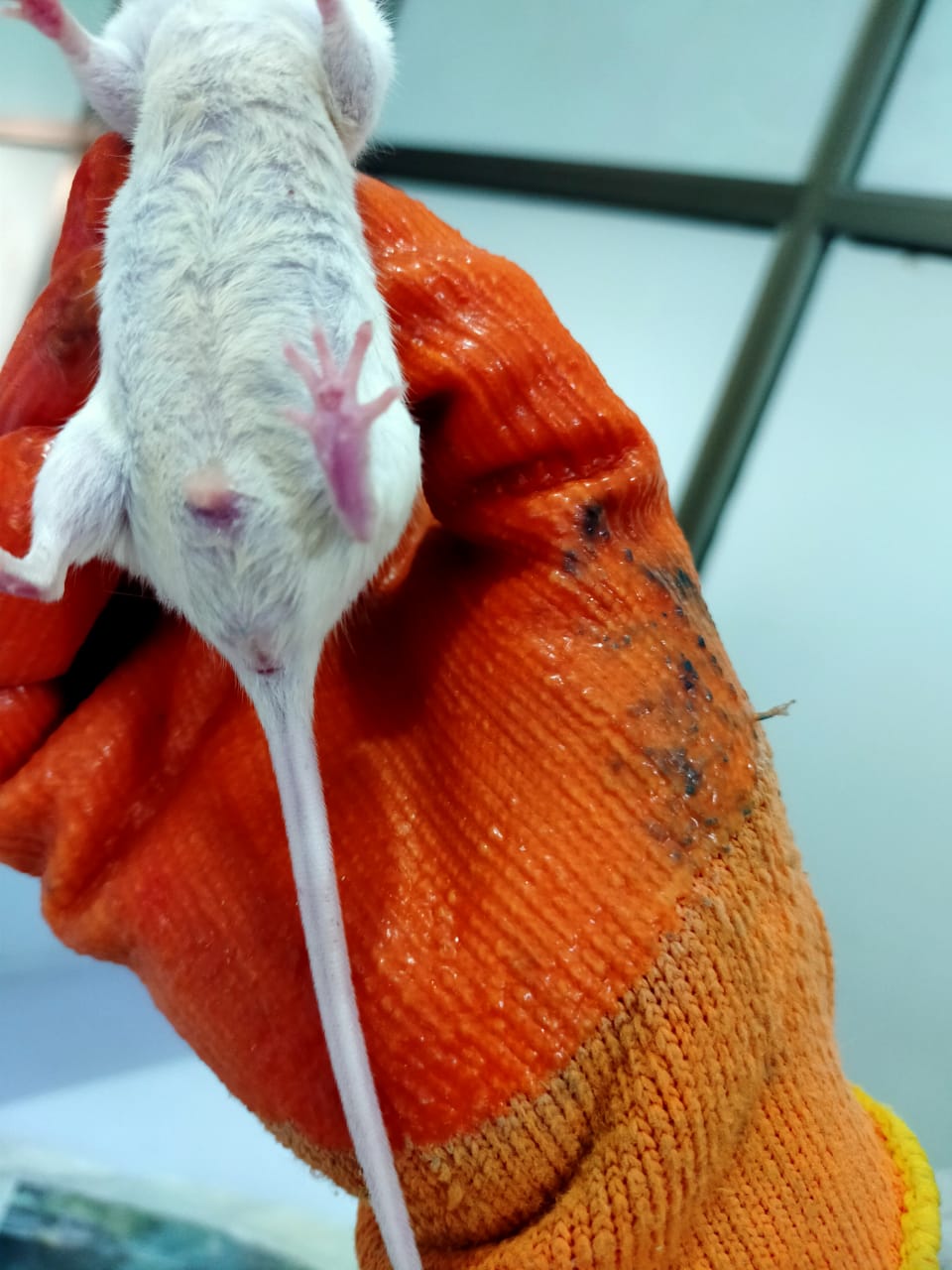

Supplement: Supplementary file 11 [file Image12.JPEG]

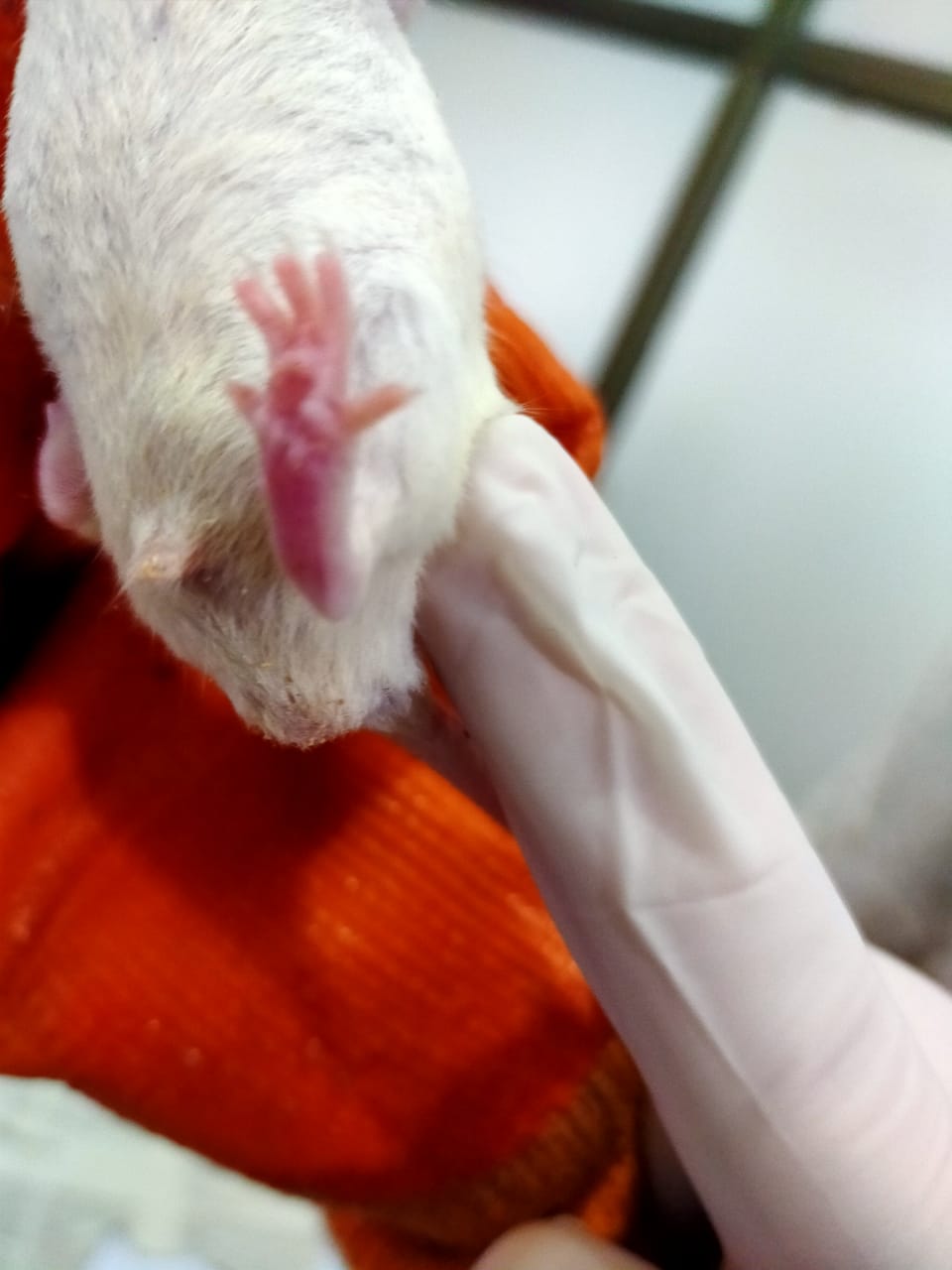

Supplement: Supplementary file 12 [file Image11.JPEG]

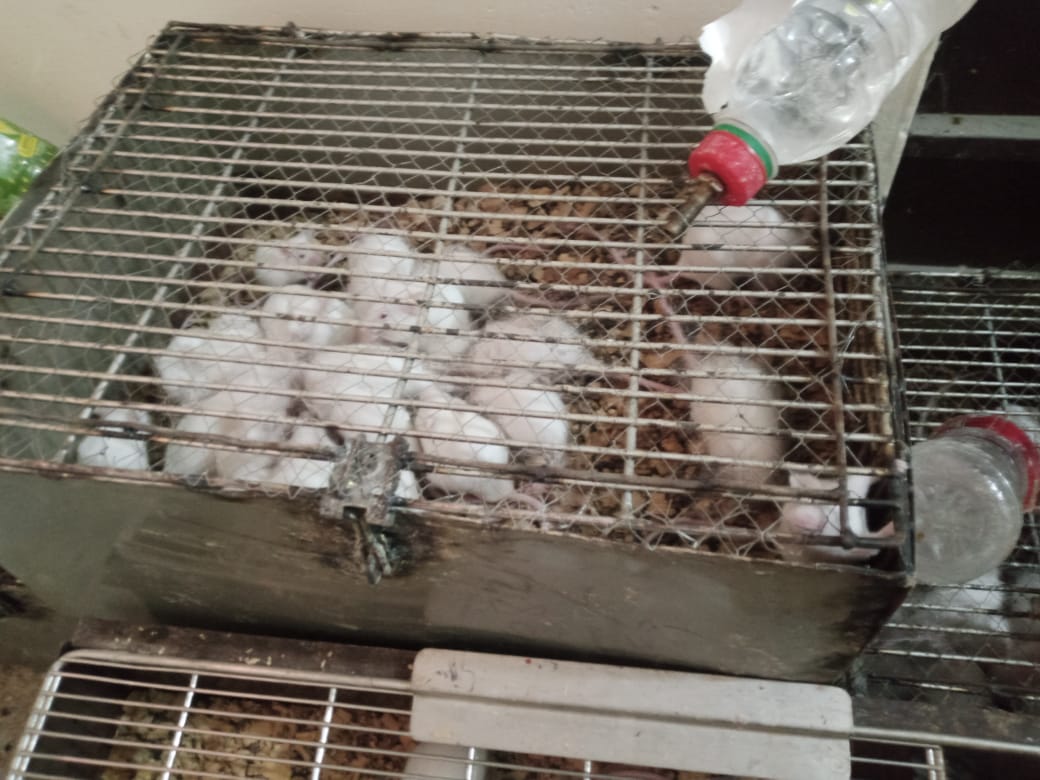

Supplement: Supplementary file 18 [file Image13.JPEG]

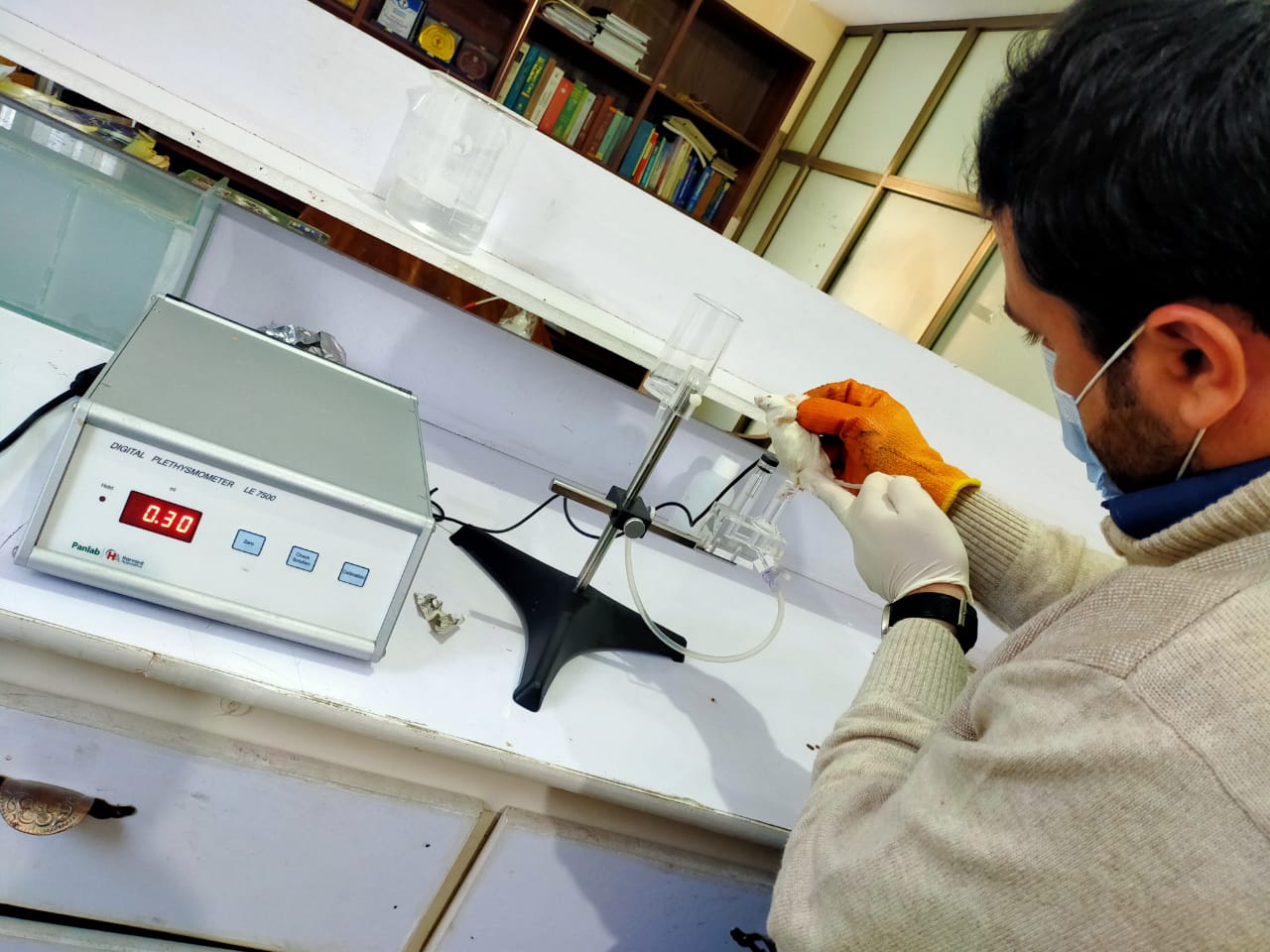

Supplement: Supplementary file 19 [file Image8.JPEG]

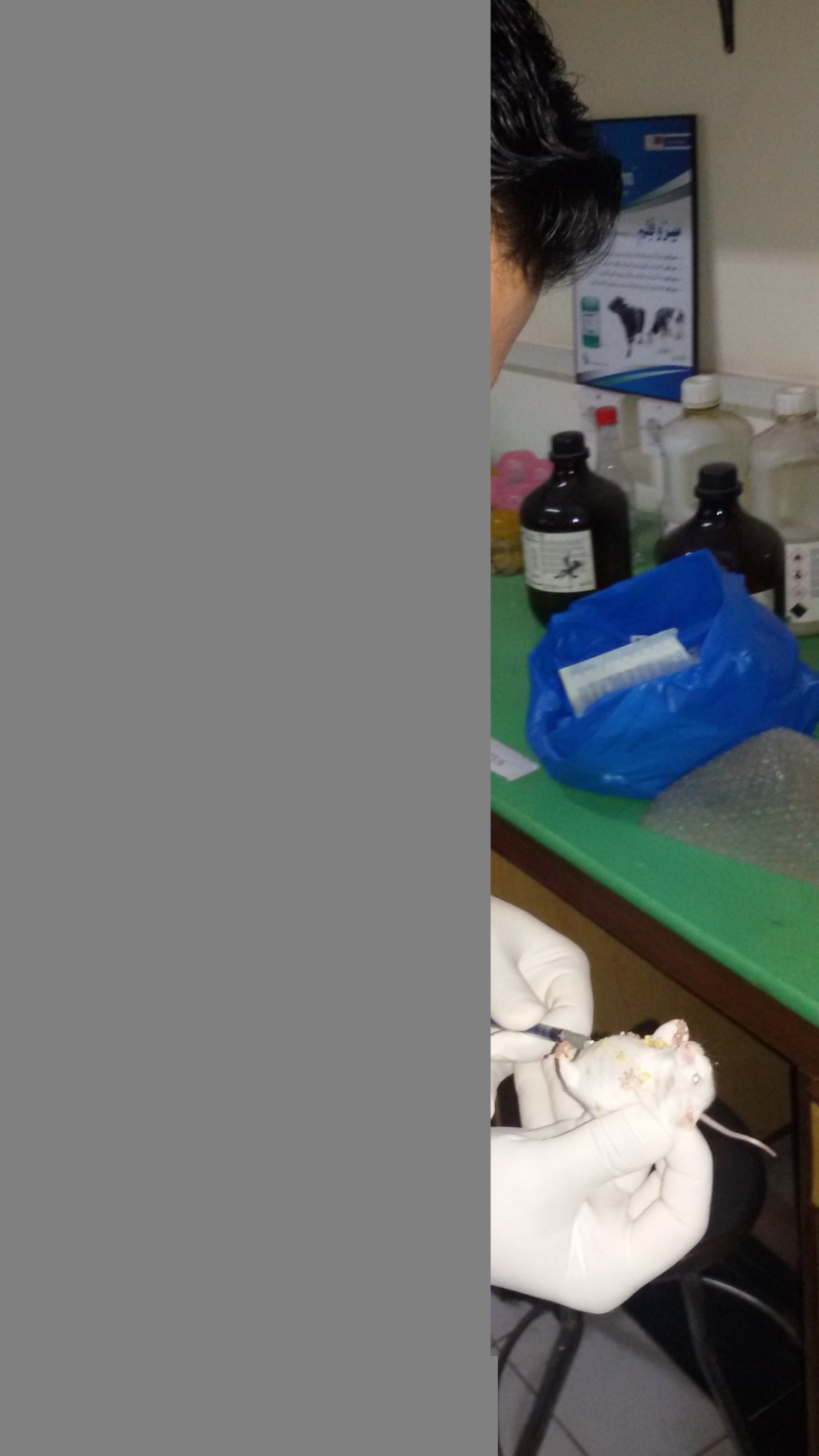

Supplement: Supplementary file 21 [file Image6.JPEG]
