# Supplementary material for: Investigation of anti-nociceptive, anti-inflammatory potential and ADMET studies of pure compounds isolated from Isodon rugosus Wall. ex Benth
Source: Front Pharmacol. 2024 Feb 13;15:1328128. doi: 10.3389/fphar.2024.1328128 (PMC10897015; doi:10.3389/fphar.2024.1328128)
Supplement: Supplementary file 14 [file Table1.DOC]

**Investigation of Anti-Nociceptive, Anti-Inflammatory Potential and ADMET Studies of Pure Compounds Isolated from *Isodon rugosus* Wall. ex Benth.**

Osama M. Alshehri^1^,, Anwar Zeb^2^, Syed Muhammad Mukarram Shah^2^, Mater H. Mahnashi^3^, Saeed Ahmed Asiri^4^, Omaish Alqahtani^5^, Abdul Sadiq^6^, Muhammad Ibrar^7^, Saleh Alshamrani^*1^, Muhammad Saeed Jan^7*^.

^1^Department of Clinical Laboratory Sciences, College of Applied Medical Sciences, Najran University, Najran, Saudi Arabia ([Omalshehri@nu.edu.sa](mailto:Omalshehri@nu.edu.sa))

^2^Department of Pharmacy, University of Swabi, Swabi, KP, Pakistan; ([zebrph@gmail.com](mailto:zebrph@gmail.com)); ([mukaramshah@uoswabi.edu.pk](mailto:mukaramshah@uoswabi.edu.pk))

^3^Department of pharmaceutical chemistry, college of pharmacy, Najran University, Najran, Kingdom of Saudi Arabia ([matermaha@gmail.com](mailto:matermaha@gmail.com))

^4^Department of Clinical Laboratory Sciences, Faculty of Applied Medical Sciences, Najran University, 1988, Najran, 61441, Saudi Arabia. ([saaasiri@nu.edu.sa](mailto:saaasiri@nu.edu.sa))

^5^Department of Pharmacognosy, College of Pharmacy, Najran University, KSA ([osalqahtani@nu.edu.sa](mailto:osalqahtani@nu.edu.sa))

^6^Department of Pharmacy, Univeristy of Malakand, Chakdara, Dir Lower, KP, Pakistan ([sadiquom@yahoo.com](mailto:sadiquom@yahoo.com))

^7^Department of Pharmacy, Bacha Khan University, Charsadda 24420, KP, Pakistan ([meet_ibrar@yahoo.com](mailto:meet_ibrar@yahoo.com))

**Corresponding Author**

*****Dr. Muhammad Saeed Jan, Department of Pharmacy, Bacha Khan University, Charsadda, KP, Pakistan. ([saeedjan@bkuc.edu.pk](mailto:saeedjan@bkuc.edu.pk)) Contact: 0092 315 3109610 & Saleh Alshamrani ([SAALSHAMRANI@NU.EDU.SA](mailto:SAALSHAMRANI@NU.EDU.SA))

**Figure S1:** ^1^H NMR spectrum of Compound 1.


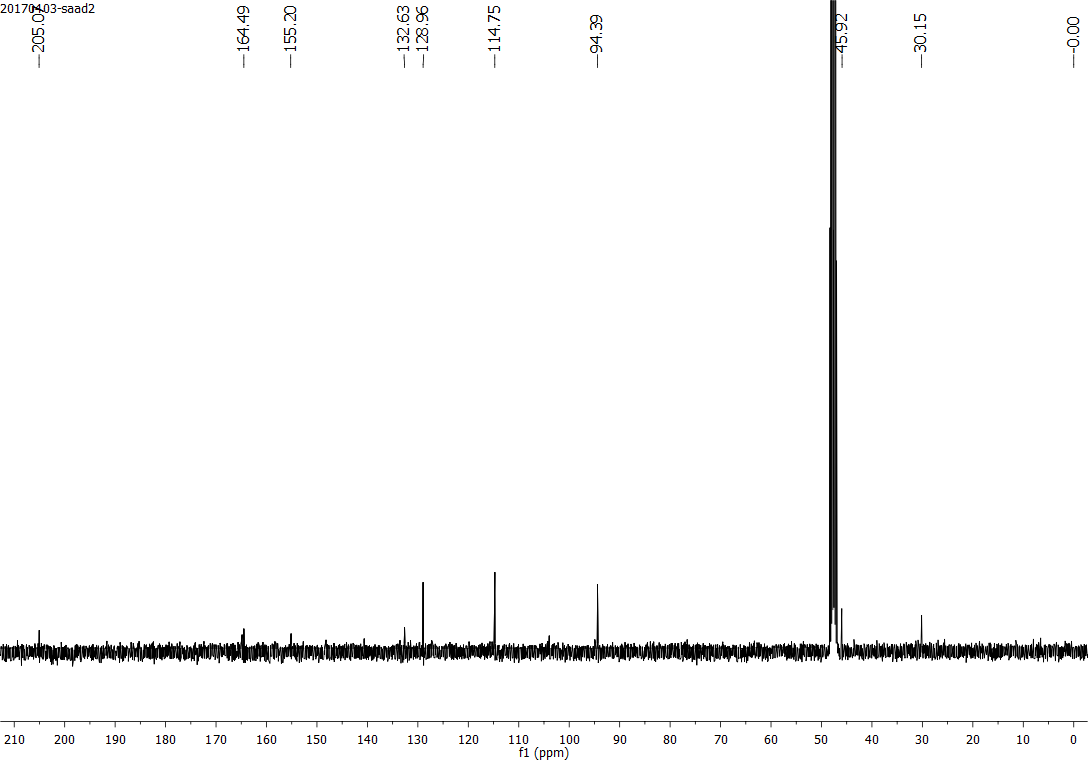


**Figure S2:** ^13^C NMR spectrum of Compound 1.


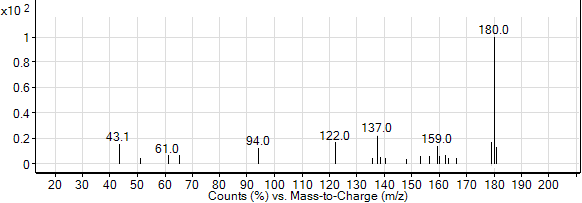


**Figure S3:** Mass spectrum of compound 1.


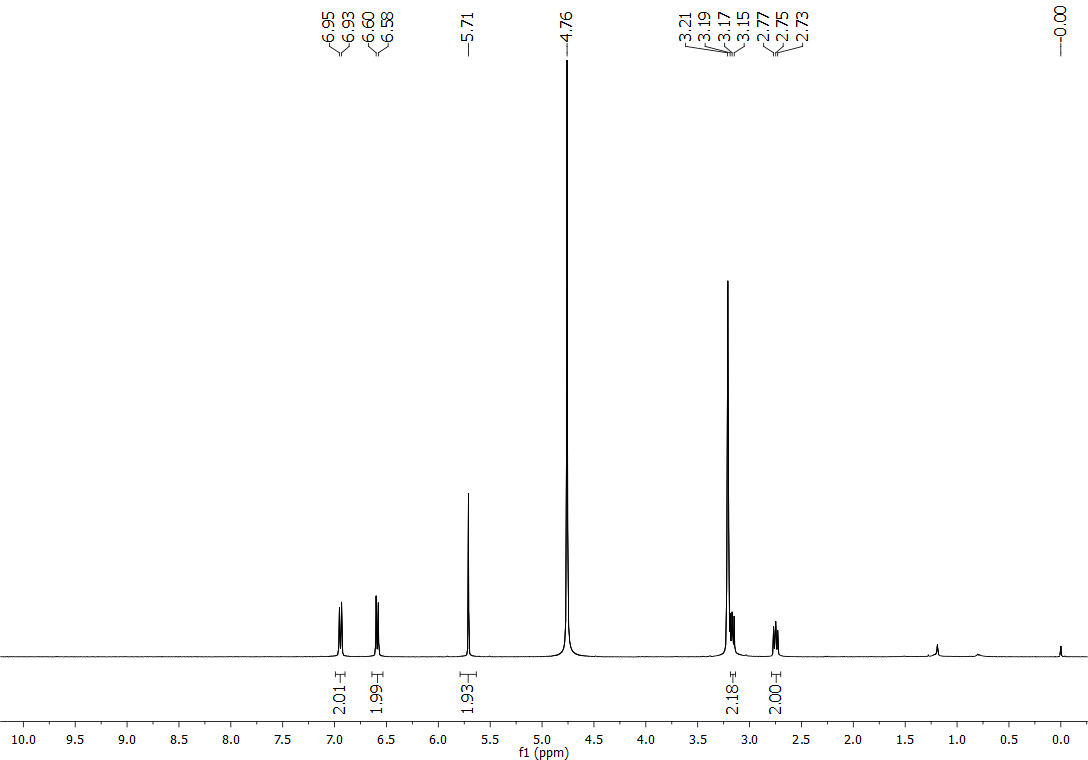


**Figure S4:** ^1^H NMR spectrum of compound 2.


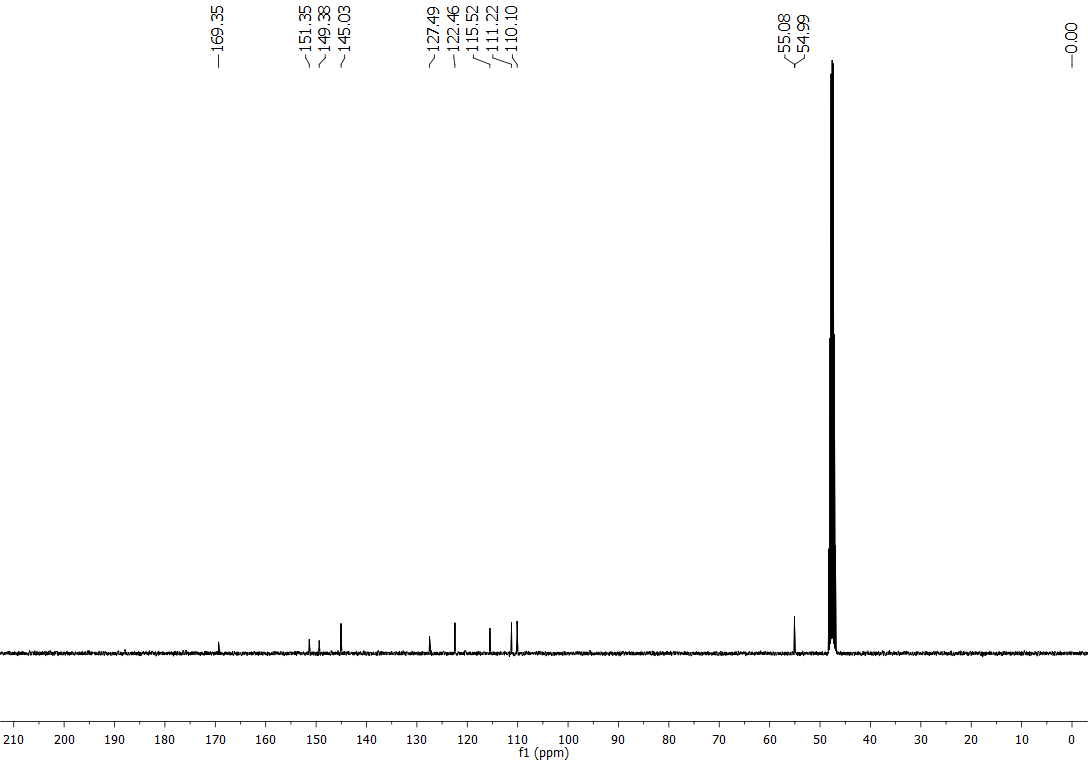


**Figure S5:** ^13^C NMR spectrum of compound 2.

**
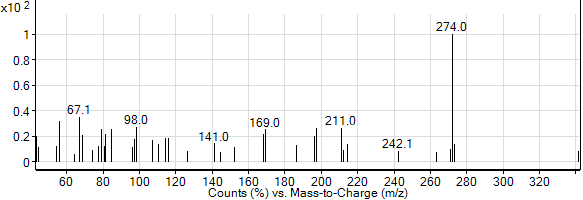
**

**Figure S6:** Mass spectrum of compound 2.
